# Impacts of Polyenvironmental Factors on DNA Methylation in Patients With Psychosis

**DOI:** 10.1093/schizbullopen/sgaf027

**Published:** 2025-11-03

**Authors:** Fatima Zahra Rami, Chaeyeong Kang, Ling Li, Thi-Hung Le, Sung-Wan Kim, Seung-Hee Won, Ariana Setiani, Byoung-Ha Yoon, Young-Chul Chung

**Affiliations:** Department of Psychiatry, Jeonbuk National University Medical School, Jeonju, Jeollabuk-do 54907, Republic of Korea; Research Institute of Clinical Medicine of Jeonbuk National University and Biomedical Research Institute of Jeonbuk National University Hospital, Jeonju, Jeollabuk-do 54907, Republic of Korea; Research Institute of Clinical Medicine of Jeonbuk National University and Biomedical Research Institute of Jeonbuk National University Hospital, Jeonju, Jeollabuk-do 54907, Republic of Korea; Department of Psychiatry, Jeonbuk National University Medical School, Jeonju, Jeollabuk-do 54907, Republic of Korea; Research Institute of Clinical Medicine of Jeonbuk National University and Biomedical Research Institute of Jeonbuk National University Hospital, Jeonju, Jeollabuk-do 54907, Republic of Korea; Department of Psychiatry, Jeonbuk National University Medical School, Jeonju, Jeollabuk-do 54907, Republic of Korea; Research Institute of Clinical Medicine of Jeonbuk National University and Biomedical Research Institute of Jeonbuk National University Hospital, Jeonju, Jeollabuk-do 54907, Republic of Korea; Department of Psychiatry, Chonnam National University Medical School, Hwasun-gun, Gwangju 58128, Republic of Korea; Department of Psychiatry, School of Medicine, Kyungpook National University, Jung-gu, Daegu 41944, Republic of Korea; Department of Psychiatry, Jeonbuk National University Medical School, Jeonju, Jeollabuk-do 54907, Republic of Korea; Research Institute of Clinical Medicine of Jeonbuk National University and Biomedical Research Institute of Jeonbuk National University Hospital, Jeonju, Jeollabuk-do 54907, Republic of Korea; Korea Research Institute of Bioscience and Biotechnology, Yuseong-gu, Daejeon 34141, Republic of Korea; Department of Psychiatry, Jeonbuk National University Medical School, Jeonju, Jeollabuk-do 54907, Republic of Korea; Research Institute of Clinical Medicine of Jeonbuk National University and Biomedical Research Institute of Jeonbuk National University Hospital, Jeonju, Jeollabuk-do 54907, Republic of Korea

**Keywords:** psychosis, DNA methylation, polyenvironmental risk score, Korea polyenvironmental risk score, differentially methylated positions, differentially methylated regions

## Abstract

**Background:**

Only a few studies have investigated the association of environmental factors with DNA methylation in schizophrenia (SZ). Our study sought to investigate differentially methylated positions (DMPs) and differentially methylated regions (DMRs) between patients with psychosis and healthy controls (HCs) and to explore associations of aberrant methylation levels with the Korea-polyenvironmental risk score-I (K-PERS-I), a comprehensive tool measuring polyenvironmental risk factors for psychosis.

**Study Design:**

Blood-based methylome-wide association study (MWAS) was conducted in patients with psychosis (*n* = 414) and HCs (*n* = 225). For MWAS, a new cutting-edge technique, Methyl-Seq was employed. Using the K-PERS-I, polyenvironmental risk factors were assessed. Psychosis-associated DMPs and DMRs were identified via beta-binomial regression, and their associations with K-PERS-I scores were examined.

**Study Results:**

We identified 1138 DMPs and 1611 DMRs associated with psychosis. In the correlation analysis, 12 DMPs-annotated genes and 11 DMRs-annotated genes were associated with childhood adversity. These genes were mainly implicated in neuronal development, neurotransmitter release, synaptic plasticity, immune response, and oxidative stress. For obstetric complications, most of top five DMPs-annotated genes were implicated in placenta function, embryonic development or gestation. For recent adult life events, top five DMPs- and DMRs-annotated genes were related to neurotransmitter production/release, oxidative stress, and stress regulation.

**Conclusions:**

We identified new psychosis-associated DMPs and DMRs. More importantly, we demonstrated how environmental factors can be biologically embedded in DNA methylation of certain genes in patients with psychosis. Ultimately, establishing causal pathways between these risk factors and DNA methylation could lead to the discovery of novel therapeutic targets.

## Introduction

Schizophrenia (SZ) is a complex mental disorder with a global incidence rate of 18.58 per 100 000 person-years,^1^ resulting in substantial personal burdens and economic costs.^2^ The underlying causes are multifactorial, involving both environmental and genetic contributions, which indicate a complex etiology. Epigenetics studies in SZ are crucial for understanding how environmental factors influence gene expression and for identifying embedded molecular signatures of environmental insults. Furthermore, altered epigenetic markers can be modified by pharmaceutical and psychosocial interventions.

To our knowledge, several systematic reviews,^3-6^ meta-analyses,^7-9^ and in silico analyses^10^^,^^11^ of DNA methylation in SZ have been published. Each reported different findings: common differentially methylated genes included *reelin*, brain-derived neurotrophic factor, dopamine, serotonin, and glutamate;^3^ several top-ranking differentially methylated positions (DMPs) were annotated to genes directly relevant to the etiology of SZ;^7^ and the top differentially methylated genes were *APC*, *CACNB2*, and *PRKN.*^10^ Regarding case-control studies that used blood samples, there have been 12 methylome-wide association studies (MWASs) in Western populations using microarray analysis^12-20^ or next-generation sequencing (NGS)-based approaches.^21-23^ The characteristics of these studies differed, and their findings were also mixed. Various thresholds were used to detect significant methylation differences, including Bonferroni correction, false discovery rate (FDR), and *P*-values such as *P* < 5 × 10^−8^, 1 × 10^−5^, or 5 × 10^−5^. Most studies reported DMPs, whereas three presented both DMPs and differentially methylated regions (DMRs).^13^^,^^14^^,^^19^ Only four studies evaluated colocalization of SZ-DMPs with SZ-associated genetic loci,^13^^,^^17^^,^^24^^,^^25^ and just five included validation analyses.^13^^,^^15^^,^^17^^,^^21^^,^^23^ Additionally, six Asian case-control studies using microarray analysis^24-29^ reported mixed findings. With the exception of one study by Li et al.,^24^ these studies had small sample sizes.

Epigenetic remodeling is influenced by environmental exposures, such as nutrition, drug intake, and physical and psychosocial factors.^30^ A broad range of environmental factors—prenatal and perinatal events, urban living, migration status, drug use, and social adversity^31^—has been associated with SZ susceptibility and may mediate risk via epigenetic modifications. Despite the potential links between environmental factors and DNA methylation levels in SZ, relatively few studies have investigated these associations. To date, 11 MWAS have explored the relationships of DNA methylation with childhood trauma,^12^^,^^32^ symptomatology,^15^^,^^16^^,^^27-29^^,^^33^^,^^34^ and other clinical phenotypes.^16^^,^^35^^,^^36^ Five tools have been developed to quantify cumulative environmental risk for SZ or psychosis using aggregate scores: the Exposome Score for SZ,^37^ polyenviromic risk score,^38^ Maudsley Environmental Risk Score,^39^ Psychosis Polyrisk Score,^40^^,^^41^ and the Korea-polyenvironmental risk score (K-PERS).^42^ We hypothesized that the impact of polyenvironmental risk factors on DNA methylation could be detected using the K-PERS-I.

In the present study, we conducted an MWAS in patients with psychosis and healthy controls (HCs) using hybridization-based NGS with the Agilent SureSelect^XT^ Human Methyl-Seq target enrichment system (Agilent Technologies, Santa Clara, CA, USA). Our aim was to identify psychosis-associated CpG loci and their annotated genes. However, the primary objective was to examine associations between aberrant DNA methylation levels and the K-PERS-I.

## Methods

### Participants

In total, 418 patients with psychosis were recruited for this study. The inclusion criteria were age between 19 and 60 years and meeting the Diagnostic and Statistical Manual of Mental Disorders, Fifth Edition^43^ criteria for SZ, schizophreniform disorder, schizoaffective disorder, psychotic disorder not otherwise specified (PNOS), delusional disorder, or brief psychotic disorder. The exclusion criteria were an intelligence quotient ≤ 70, history of head trauma, serious neurological disorders, and significant medical illness. Age-, sex-, and education-matched HCs were recruited via advertisements. All participants provided written informed consent in accordance with a protocol approved by the Ethics Committee of Jeonbuk National University Hospital (approval no. CUH 2014-11-002).

### Clinical Assessment

The severity of symptoms was evaluated within one week of blood sampling using the positive and negative syndrome scale (PANSS).^44^ Environmental risk factors associated with SZ were measured using the K-PERS-I.^42^ This instrument includes six domain scores: childhood adversity, obstetric complications, paternal age at birth, parental socioeconomic status (pSES), recent adult life events, and urbanicity, along with a total score (see Table S1). Chlorpromazine (CPZ) dose equivalents were calculated using the defined daily doses method.^45^

### DNA Methylation Assay

A flowchart of the experimental procedures is presented in Figure S1. DNA was extracted from peripheral blood mononuclear cell samples. After assessing quality, the DNA samples were fragmented to a median size of 150 base pairs. After hybridization, bisulfite conversion, and polymerase chain reaction, methylation sequencing was performed using an Illumina NovaSeq 6000 system (Illumina, San Diego, CA, USA). The SureSelect^XT^ Human Methyl-Seq panel covers over 3.7 million CpG sites across the genome with 100× coverage (Agilent Technologies).

### Data Processing and Statistical Modeling

After trimming and quality filtering, reads were mapped to the human reference genome GRCh38/hg38. Duplicate reads were removed, and methylation calls were made using the Bismark methylation extractor. Single-nucleotide polymorphisms with a minor allele frequency ≥ 5% were excluded, and principal component analysis (PCA) was performed using deepTools (v.3.5.1, https://github.com/deeptools/deepTools) to identify and remove outliers.

The raw methylation values of each CpG site were modeled using beta-binomial regression with an arcsine link function.^46^ To control for age, sex, batch effects, cell heterogeneity, and PCA components (to account for unmeasured confounding factors), these variables were included as covariates in the regression model using the assocComp function in the methylKit package.^47^ Psychosis-associated DMPs were defined by a family-wise error rate (FWER)-adjusted *P*-value < .05 and a methylation difference ≥ 3% between patients and HCs.^48-50^ Psychosis-associated DMRs were identified using comb-p^51^ with a maximum distance of 500 bp and a seeded *P*-value of .001. Differentially methylated region analyses included all significant CpG sites; regions with at least three probes and a Sidak-corrected *P*-value < .05 were considered significant. These parameters are consistent with previous work.^52^ The identified DMPs and DMRs were annotated using Hypergeometric Optimization of Motif EnRichment (HOMER),^53^ which determines the distance of a DMR to the nearest transcription start site and assigns the DMR to that gene.

### Gene Ontology Enrichment Analysis

Gene Ontology (GO) analysis was performed using Enrichr (https://maayanlab.cloud/Enrichr/). Pathways with an FDR < 0.05 were considered statistically significant.

### Correlation Analysis

Pearson correlation analysis was performed to examine associations between psychosis-associated DMPs (1138 sites) and DMRs (1611 regions) and K-PERS-I scores. Multiple comparisons (*n* = 6) were corrected using the FWER. All analyses were adjusted for age, sex, duration of illness (DI), and CPZ equivalent dose. Because we hypothesized that indirect and nonspecific environmental factors such as paternal age at birth, pSES, urbanicity, and the total K-PERS-I score were unlikely to have substantial and specific effects on DNA methylation, we focused on childhood adversity, obstetric complications, and recent adult life events as the main outcomes of interest.

### Overlapping with SZ-associated MWAS Genes

We compared our top 50 psychosis-associated DMP- and DMR-annotated genes with those reported in previous MWAS of SZ or psychosis using genome-wide DNA methylation in blood samples.^7^^,^^12-15^^,^^17^^,^^19^^,^^20^^,^^22^^,^^24^^,^^26-29^^,^^54^^,^^55^

### Overlapping with SZ-associated GWAS Genes

We also compared our top 50 psychosis-associated DMP- and DMR-annotated genes with those identified in genome-wide association studies (GWAS) of SZ conducted in Asian populations^56-58^ and European populations.^59^

## Results

### Demographic and Clinical Characteristics of the Participants

Given that four outliers detected by PCA were removed, a total of 414 individuals diagnosed with psychosis and 225 HCs were included in the analysis. The patient diagnoses were heterogeneous: SZ (*n* = 273), schizoaffective disorder (*n* = 5), schizophreniform disorder (*n* = 56), PNOS (*n* = 70), delusional disorder (*n* = 6), and brief psychotic disorder (*n* = 4). Additionally, there were significant differences in education level and several K-PERS-I scores between the two groups (*P* < .001) (Table 1).

**Table 1:** Demographic and Clinical Characteristics of the Participants

	**Patients (*n* = 414)**	**HCs (*n* = 225)**	***P*-value**
**Age (years)**	36.27 ± 11.83 (*n* = 414)	37.07 ± 11.90 (*n* = 225)	.416
**Sex**			.259
**Male (%)**	189 (46%)	114 (51%)	
**Female (%)**	225 (54%)	111 (49%)	
**Edu (years)**	10.63 ± 1.61 (*n* = 414)	14.13 ± 2.50 (*n* = 225)	<.001
**DI (months)**	120.68 ± 124.44 (*n* = 399)		
**CPZ equivalent**	411.14 ± 332.53 (*n* = 291)^a^		
**PANSS**			
**Positive symptoms**	14.12 ± 7.30 (*n* = 414)		
**Negative symptoms**	12.37 ± 7.01 (*n* = 414)		
**General psychopathology**	28.51 ± 9.65 (*n* = 414)		
**Total PANSS**	55.98 ± 20.24 (*n* = 414)		
**K-PERS-I**			
**Childhood adversity**	3.41 ± 1.93 (*n* = 346)	0.96 ± 1.85 (*n* = 224)	<.001
**Obstetric complications**	0.13 ± 0.50 (*n* = 313)	0.06 ± 0.35 (*n* = 224)	.051
**Paternal age at birth**	−0.24 ± 0.44 (*n* = 349)	−0.36 ± 0.35 (*n* = 223)	<.001
**Parental SES**	0.43 ± 0.44 (*n* = 351)	0.37 ± 0.48 (*n* = 222)	.203
**Recent adult life events**	4.50 ± 1.58 (*n* = 298)	−0.03 ± 3.31 (*n* = 225)	<.001
**Urbanicity**	−1.18 ± 1.47 (*n* = 350)	−0.94 ± 1.40 (*n* = 185)	.061
**Total score**	6.95 ± 3.33 (*n* = 259)	−0.17 ± 4.27 (*n* = 184)	<.001
**Diagnosis (%)**			
**Schizophrenia**	273 (66%)		
**Schizoaffective disorder**	5 (1%)		
**Schizophreniform disorder**	56 (14%)		
**Psychotic disorder NOS**	70 (17%)		
**Delusional disorder**	6 (1%)		
**Brief psychotic disorder**	4 (1%)		

a Among 414, 78 were antipsychotic-free and 45 antipsychotic-naïve.

Abbreviations: Mean ± SD, CPZ, chlorpromazine; DI, duration of illness; Edu, education; HCs, healthy controls; K-PERS, Korea polyenvironmental risk score; NOS, not otherwise specified; PANSS: positive and negative syndrome scale; SES: socio-economic status.

### Psychosis-associated DMPs

**Figure 1:**
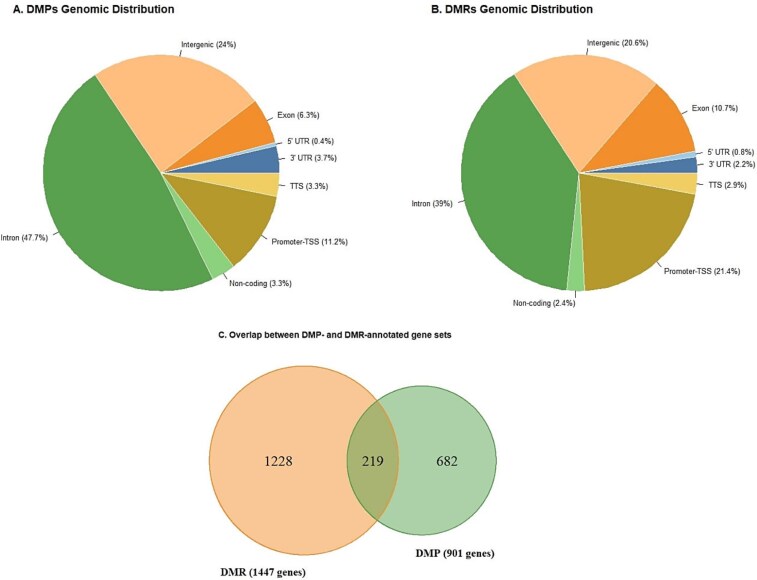
(A) Genomic distribution of DMPs, (B) genomic distribution of DMRs, (C) Venn diagram showing the overlap between DMP- and DMR-annotated gene sets. The data presented include 1138 significant DMPs after FWER correction and 1611 significant DMRs after Sidak correction. Abbreviations: FWER, family-wise error rate; TSS, transcription start site; TTS, transcription termination site; UTR, untranslated region; DMPs, differentially methylated positions; DMRs, differentially methylated regions.

In total, 1138 of 1 597 844 CpG sites that passed quality control had an adjusted *P*-value < .05. Among these sites, 611 were hypermethylated, whereas 527 were hypomethylated. The majority of DMPs were located in intronic, intergenic, and promoter-TSS regions (Figure 1A). These DMPs were mapped to 901 unique genes. The top 50 DMPs and their annotated genes are presented in Table 2.

**Table 2:** Top 50 DMPs Between Patients with Psychosis and HCs

**Chr**	**BP**	**Methylation difference**	***P*-value**	**FWER**	**Annotation**	**Distance to TSS**	**Gene name**	**Methylation status**	**Overlapping with Asian SZ-SNPs** ^**a**^	**Overlapping with European SZ-SNPs** ^**b**^
9	131 289 107	7.01	1.07 × 10^−104^	1.71 × 10^−98^	Promoter-TSS	−616	PLPP7	Hyper	NO	NO
9	131 289 068	6.20	2.40 × 10^−82^	3.83 × 10^−76^	Promoter-TSS	−655	PLPP7	Hyper	NO	NO
11	6 497 556	−8.30	4.17 × 10^−80^	6.66 × 10^−74^	Intron	260	DNHD1	Hypo	NO	NO
22	26 466 188	8.79	1.89 × 10^−73^	3.01 × 10^−67^	Intron	13 300	HPS4	Hyper	NO	NO
11	126 408 181	−7.38	5.66 × 10^−71^	9.05 × 10^−65^	Exon	2094	ST3GAL4	Hypo	NO	NO
18	46 753 170	8.06	1.24 × 10^−67^	1.99 × 10^−61^	Intron	3883	ST8SIA5	Hyper	NO	NO
9	87 859 362	7.01	1.74 × 10^−62^	2.78 × 10^−56^	Promoter-TSS	−306	LOC392364	Hyper	NO	NO
11	72 989 735	−8.89	2.63 × 10^−61^	4.21 × 10^−55^	Intron	152 583	FCHSD2	Hypo	NO	NO
4	8 093 419	7.30	1.13 × 10^−60^	1.81 × 10^−54^	Intron	−21 431	ABLIM2	Hyper	NO	NO
9	97 396 179	−7.67	4.72 × 10^−56^	7.54 × 10^−50^	Intron	512	LOC286359	Hypo	NO	NO
1	156 559 988	−7.17	5.07 × 10^−54^	8.10 × 10^−48^	Intron	12 577	IQGAP3	Hypo	NO	NO
7	55 260 827	−7.17	2.37 × 10^−53^	3.79 × 10^−47^	Intergenic	−5192	ELDR	Hypo	NO	NO
8	141 229 279	7.39	3.47 × 10^−53^	5.54 × 10^−47^	Promoter-TSS	−705	SLC45A4	Hyper	NO	NO
8	11 853 551	−8.23	2.62 × 10^−51^	4.18 × 10^−45^	Intron	14 536	CTSB	Hypo	NO	NO
4	3 263 984	7.60	3.73 × 10^−50^	5.96 × 10^−44^	Intergenic	14 825	MSANTD1	Hyper	NO	NO
17	29 717 084	5.31	6.27 × 10^−50^	1.00 × 10^−43^	Intron	44 348	SSH2	Hyper	NO	NO
7	1 312 125	−6.71	1.44 × 10^−49^	2.30 × 10^−43^	Intergenic	79 253	UNCX	Hypo	NO	NO
1	14 929 641	5.51	9.67 × 10^−49^	1.55 × 10^−42^	Promoter-TSS	−25	KAZN	Hyper	NO	NO
18	9 478 797	8.21	4.99 × 10^−48^	7.98 × 10^−42^	Intron	3284	RALBP1	Hyper	NO	NO
1	21 518 330	5.72	5.49 × 10^−46^	8.77 × 10^−40^	Intron	8907	ALPL	Hyper	NO	NO
17	15 258 278	6.76	1.00 × 10^−45^	1.60 × 10^−39^	Intron	2542	PMP22	Hyper	NO	NO
8	141 232 806	5.14	2.03 × 10^−45^	3.25 × 10^−39^	Intron	−4232	SLC45A4	Hyper	NO	NO
4	151 666 819	−6.31	2.48 × 10^−45^	3.96 × 10^−39^	3’ UTR	94 188	GATB	Hypo	NO	NO
18	59 138 961	−5.32	2.73 × 10^−44^	4.37 × 10^−38^	Promoter-TSS	−923	SEC11C	Hypo	NO	NO
11	125 607 484	−5.95	1.20 × 10^−43^	1.91 × 10^−37^	Intron	14 632	STT3A	Hypo	NO	NO
13	37 105 593	6.37	1.44 × 10^−43^	2.30 × 10^−37^	Promoter-TSS	71	CSNK1A1L	Hyper	NO	NO
10	17 305 362	−7.26	5.00 × 10^−41^	7.98 × 10^−35^	Intergenic	−75 377	VIM-AS1	Hypo	NO	NO
5	31 407 750	6.05	2.55 × 10^−40^	4.07 × 10^−34^	Intron	124 343	DROSHA	Hyper	NO	NO
16	2 913 528	5.51	4.58 × 10^−40^	7.31 × 10^−34^	Intron	1549	FLYWCH1	Hyper	NO	NO
12	107 685 059	5.24	9.21 × 10^−40^	1.47 × 10^−33^	Promoter-TSS	−673	PWP1	Hyper	NO	NO
7	1 312 167	−5.84	1.17 × 10^−39^	1.87 × 10^−33^	Intergenic	79 295	UNCX	Hypo	NO	NO
6	157 593 224	−5.95	4.26 × 10^−39^	6.80 × 10^−33^	Intron	64 092	MIR3692	Hypo	NO	NO
6	153 059 300	−5.33	5.08 × 10^−39^	8.12 × 10^−33^	Intron	−56 485	MTRF1L	Hypo	NO	NO
4	1 555 854	4.56	1.25 × 10^−38^	1.99 × 10^−32^	Intergenic	128 137	FAM53A	Hyper	NO	NO
3	16 978 933	9.39	1.35 × 10^−37^	2.16 × 10^−31^	Intron	45 737	MIR3714	Hyper	NO	NO
11	130 881 414	5.19	6.51 × 10^−37^	1.04 × 10^−30^	Intron	−11 167	LOC103611081	Hyper	NO	NO
2	237 858 123	−6.20	9.43 × 10^−37^	1.51 × 10^−30^	Intergenic	−1500	RAMP1	Hypo	NO	NO
11	134 977 635	4.86	1.97 × 10^−36^	3.15 × 10^−30^	Intergenic	−7717	LOC100507548	Hyper	NO	NO
14	103 141 017	7.00	3.06 × 10^−36^	4.89 × 10^−30^	Intergenic	14 711	TNFAIP2	Hyper	NO	NO
1	236 539 861	−6.50	4.74 × 10^−36^	7.58 × 10^−30^	Intron	−15 353	LGALS8-AS1	Hypo	NO	NO
18	58 124 212	−6.69	6.65 × 10^−36^	1.06 × 10^−29^	Intron	−25 108	NEDD4L	Hypo	NO	NO
8	118 108 171	−5.26	1.64 × 10^−35^	2.62 × 10^−29^	Intron	3648	EXT1	Hypo	NO	NO
6	168 279 697	−6.15	5.65 × 10^−35^	9.03 × 10^−29^	Intergenic	36 759	LOC101929420	Hypo	NO	NO
13	109 310 072	−5.22	1.05 × 10^−34^	1.68 × 10^−28^	Intergenic	−90 624	LINC00399	Hypo	NO	NO
1	161 958 213	6.61	1.18 × 10^−34^	1.89 × 10^−28^	Intron	66 252	OLFML2B	Hyper	NO	NO
3	52 794 613	3.80	5.08 × 10^−34^	8.12 × 10^−28^	Promoter-TSS	−155	ITIH3	Hyper	NO	YES
12	107 685 147	5.57	7.45 × 10^−33^	1.19 × 10^−26^	Promoter-TSS	−585	PWP1	Hyper	NO	NO
6	167 294 272	5.15	9.58 × 10^−33^	1.53 × 10^−26^	Intron	2957	UNC93A	Hyper	NO	NO
12	124 501 973	6.38	1.31 × 10^−32^	2.09 × 10^−26^	Intron	65 639	NCOR2	Hyper	NO	NO
16	29 155 817	−4.47	5.96 × 10^−32^	9.52 × 10^−26^	Intergenic	80 975	RRN3P2	Hypo	NO	NO

Corresponding genes of previously reported SNPs associated SZ in Asian populations^56^^-^^58^ and European populations.^59^

Abbreviations: BP, base pair; Chr, chromosome; DMPs, differentially methylated positions; FWER, family wise error rate; HCs, healthy controls; Hyper, hypermethylation; Hypo, hypomethylation; SNP, single-nucleotide polymorphism; SZ, schizophrenia; TSS, transcription start site; UTR, untranslated region.

### Psychosis-associated DMRs

Differentially methylated regions were identified by combining information from nearby CpG sites. Using comb-p, we detected 1611 genomic regions with a significant Sidak-adjusted *P*-value, each containing a minimum of three CpG sites. Most DMRs were located in intronic, promoter-TSS, intergenic, and exon regions (Figure 1B). A total of 219 genes were shared between the DMP- and DMR-annotated gene sets (Figure 1C). In total, these DMRs overlapped with 1447 unique genes. The top 50 DMRs and their annotated genes are presented in Table 3.

**Table 3:** Top 50 DMRs Between Patients with Psychosis and HCs

**Chr**	**Start**	**End**	***N* probes**	***P*-value**	**Šidák p**	**Annotation**	**Distance to TSS**	**Gene Name**	**Overlapping with Asian SZ-SNPs** ^**a**^	**Overlapping with European SZ-SNPs** ^**b**^
12	107 684 808	107 685 179	7	7.18 × 10^−45^	1.24 × 10^−42^	Promoter-TSS	−738	PWP1	NO	NO
17	76 274 036	76 274 102	5	1.30 × 10^−45^	1.26 × 10^−42^	TTS	8721	UBALD2	NO	NO
12	9 943 261	9 943 752	22	5.26 × 10^−25^	6.87 × 10^−23^	Promoter-TSS	−12	LINC02470	NO	NO
4	3 293 814	3 293 847	3	1.84 × 10^−25^	3.58 × 10^−22^	Intergenic	−20 316	RGS12	NO	NO
22	20 424 012	20 424 211	13	5.43 × 10^−24^	1.75 × 10^−21^	TTS	13 713	SCARF2	NO	NO
16	29 155 648	29 155 846	4	4.12 × 10^−23^	1.34 × 10^−20^	Intergenic	80 905	RRN3P2	NO	NO
4	56 657 541	56 657 728	3	2.56 × 10^−22^	8.80 × 10^−20^	Intron	−1113	HOPX	NO	NO
5	31 470 750	31 470 868	4	2.60 × 10^−22^	1.42 × 10^−19^	Intron	61 284	DROSHA	NO	NO
3	160 026 399	160 026 567	3	5.95 × 10^−22^	2.27 × 10^−19^	Intron	5834	LINC01100	NO	NO
12	54 369 346	54 369 585	14	6.15 × 10^−21^	1.65 × 10^−18^	3’ UTR	−4980	GPR84	NO	NO
19	4 518 811	4 518 937	8	4.98 × 10^−21^	2.53 × 10^−18^	Promoter-TSS	−388	PLIN4	NO	NO
2	138 781 177	138 781 638	15	7.73 × 10^−20^	1.08 × 10^−17^	Intergenic	−1018	NXPH2	NO	NO
8	11 862 297	11 862 856	10	8.72 × 10^−19^	1.00 × 10^−16^	Intron	5510	CTSB	NO	NO
10	129 871 075	129 871 371	13	4.82 × 10^−19^	1.05 × 10^−16^	Intron	−27 849	MIR4297	NO	NO
4	3 373 144	3 373 419	11	5.17 × 10^−19^	1.21 × 10^−16^	Intron	3084	RGS12	NO	NO
7	4 204 638	4 204 932	16	6.39 × 10^−19^	1.40 × 10^−16^	Intron	−69 948	LOC105375131	NO	NO
12	110 012 839	110 012 920	4	1.83 × 10^−19^	1.45 × 10^−16^	Intron	13 450	ANKRD13A	NO	NO
8	140 558 499	140 558 772	11	9.41 × 10^−19^	2.21 × 10^−16^	Intron	46 640	CHRAC1	NO	NO
4	108 165 098	108 166 320	23	5.72 × 10^−17^	5.88 × 10^−15^	Intron	1088	LEF1	NO	NO
1	3 352 635	3 352 980	16	1.45 × 10^−16^	2.07 × 10^−14^	Intron	−101 857	ARHGEF16	NO	NO
15	60 479 571	60 479 897	5	1.87 × 10^−16^	4.37 × 10^−14^	Promoter-TSS	556	RORA-AS1	NO	NO
1	153 609 729	153 610 209	8	4.42 × 10^−16^	5.94 × 10^−14^	Promoter-TSS	−614	S100A16	NO	NO
17	12 522 451	12 522 559	4	2.10 × 10^−16^	1.32 × 10^−13^	Intergenic	−27 463	LINC00670	NO	NO
11	59 173 856	59 173 995	6	3.74 × 10^−16^	1.54 × 10^−13^	Intron	1794	DTX4	NO	NO
5	75 320 839	75 320 927	3	1.68 × 10^−16^	1.62 × 10^−13^	Intergenic	−16 285	HMGCR	NO	NO
1	161 605 835	161 606 241	26	1.30 × 10^−15^	2.11 × 10^−13^	Promoter-TSS	−21	HSPA7	NO	NO
9	122 225 828	122 226 927	74	3.73 × 10^−15^	2.20 × 10^−13^	Exon	1163	LHX6	NO	NO
10	133 527 731	133 528 689	69	5.05 × 10^−15^	3.35 × 10^−13^	Intron	847	CYP2E1	NO	NO
9	120 842 075	120 842 288	11	1.47 × 10^−15^	4.35 × 10^−13^	Promoter-TSS	740	PSMD5	NO	NO
11	120 169 724	120 169 910	10	1.32 × 10^−15^	4.60 × 10^−13^	Noncoding	840	LOC107984399	NO	NO
22	50 578 522	50 578 759	12	1.77 × 10^−1^5	4.81 × 10^−13^	Promoter-TSS	−29	CPT1B	NO	NO
2	202 035 809	202 036 749	43	7.77 × 10^−15^	5.31 × 10^−13^	Exon	1692	FZD7	NO	NO
20	62 831 112	62 831 902	14	6.71 × 10^−15^	5.41 × 10^−13^	Intron	−13 059	DPH3P1	NO	NO
2	3 780 627	3 780 815	9	1.71 × 10^−15^	5.68 × 10^−13^	Intron	73 879	DCDC2C	NO	NO
5	177 338 987	177 339 517	9	4.95 × 10^−15^	6.05 × 10^−13^	Intron	12 416	LMAN2	NO	NO
8	23 304 587	23 304 872	11	2.77 × 10^−15^	6.25 × 10^−13^	Intron	16 622	R3HCC1	NO	NO
1	182 301 253	182 301 383	6	2.95 × 10^−15^	1.48 × 10^−12^	Intron	12 743	LINC01344	NO	NO
7	64 885 013	64 885 131	3	2.73 × 10^−15^	1.51 × 10^−12^	Intergenic	−18 170	ZNF273	NO	NO
7	76 499 870	76 500 119	13	5.83 × 10^−15^	1.52 × 10^−−12^	Intron	−10 428	UPK3B	NO	NO
11	1 849 921	1 850 003	5	2.03 × 10^−15^	1.56 × 10^−12^	Intergenic	−3122	LSP1	NO	NO
12	132 178 779	132 179 204	20	2.93 × 10^−14^	4.43 × 10^−12^	Intergenic	10 703	LINC02361	NO	NO
6	1 515 413	1 515 492	3	7.35 × 10^−15^	5.95 × 10^−12^	Intergenic	−90 078	FOXCUT	NO	NO
16	29 139 979	29 140 604	3	7.63 × 10^−14^	7.83 × 10^−12^	Intergenic	65 450	RRN3P2	NO	NO
4	1 555 798	1 555 932	6	1.75 × 10^−14^	8.40 × 10^−12^	Intergenic	128 126	FAM53A	NO	NO
2	201 985 802	201 985 880	4	1.06 × 10^−14^	8.77 × 10^−12^	Intergenic	−48 746	FZD7	NO	NO
4	646 489	646 834	20	5.76 × 10^−14^	1.07 × 10^−11^	Intron	−6515	PDE6B	NO	NO
3	149 376 867	149 377 527	14	2.15 × 10^−13^	2.09 × 10^−11^	Promoter-TSS	452	TM4SF1	NO	NO
7	100 813 972	100 814 084	7	4.18 × 10^−14^	2.40 × 10^−11^	Intron	−12 841	SLC12A9	NO	NO
6	168 132 648	168 133 048	21	2.29 × 10^−13^	3.68 × 10^−11^	Intergenic	−51 291	FRMD1	NO	NO
7	77 328 389	77 328 848	6	3.08 × 10^−13^	4.31 × 10^−11^	Exon	−86 579	LOC101927243	NO	NO

Šidák *P*-values represent *P*-values after multiple testing corrections, with a minimum of three probes. Parameters for Comb-p were Dist = 500 and threshold 0.001.

Corresponding genes of previously reported SNPs associated SZ in Asian populations^56^^-^^58^ and European populations.^59^

Abbreviations: Chr, chromosome; DMRs, differentially methylated regions; HCs, healthy controls; SNP, single-nucleotide polymorphism; SZ, schizophrenia; TSS, transcription start site; TTS, transcription termination site; UTR, untranslated region.

### GO Enrichment Analyses

Genes mapped to DMPs and DMRs were used to perform GO analysis. Several biological processes showed enrichment at the nominal (unadjusted) significance level (*P* < .05); however, none remained significant after FDR correction.

### Correlation of DMP Methylation Levels with K-PERS-I

In total, 12 DMP-annotated genes exhibited significant correlations with childhood adversity. The top 10 genes were *NRADDP* (adjusted *P* = .002), *LINC01605* (adjusted *P* = .005), *SOX8* (adjusted *P* = .005), *PIK3CD* (adjusted *P* = .009), *FZD10-AS1* (adjusted *P* = .022), *LINC02222* (adjusted *P* = .028), *SCN10A* (adjusted *P* = .032), *CTSB* (adjusted *P* = .038), *FAM53B-AS1* (adjusted *P* = .039), and *LINC01983* (adjusted *P* = .043). Six DMP-annotated genes were significantly correlated with obstetric complications. The top five genes were *PFKFB3* (adjusted *P* = .009), *NMNAT3* (adjusted *P* = .026), *PAK2* (adjusted *P* = .033), *APCDD1L* (adjusted *P* = .034), and *RASGRP3* (adjusted *P* = .036). Eleven DMP-annotated genes exhibited significant associations with recent adult life events, of which the top five were *DPEP1* (adjusted *P* = .002), *GABARAPL1* (adjusted *P* = .003), *ADGRG5* (adjusted *P* = .019), *MTR* (adjusted *P* = .020), and *EMC2* (adjusted *P* = .022) (Table 4). Results for other subdomains are provided in Table S1.

**Table 4:** Correlation Between the Methylation Level of DMPs and K-PERS-I

**Subdomains of** **the K-PERS-I**	**Chr**	**Position (bp)**	***r***	***P*-value**	**FWER**	**Gene**	**Genomic location distribution**
**Childhood adversity**							
	3	47 001 749	0.19	<.001	0.002	NRADDP	Exon
	8	37 517 941	−0.18	.001	0.005	LINC01605	Intron
	16	1 020 029	0.18	.001	0.005	SOX8	Intergenic
	1	9 734 248	0.17	.001	0.009	PIK3CD	Intron
	12	130 138 965	0.16	.004	0.022	FZD10-AS1	Intergenic
	5	180 684 145	0.15	.005	0.028	LINC02222	Promoter-TSS
	3	38 823 447	0.15	.005	0.032	SCN10A	Intergenic
	8	11 915 156	−0.15	.006	0.038	CTSB	Intergenic
	10	124 626 167	0.15	.006	0.039	FAM53B-AS1	Intron
	3	195 860 229	0.15	.007	0.043	LINC01983	Intron
**Obstetric complications**							
	10	6 178 247	−0.18	.002	0.009	PFKFB3	Intron
	3	139 679 060	0.16	.004	0.026	NMNAT3	Intergenic
	3	196 738 942	0.16	.005	0.033	PAK2	Promoter-TSS
	20	58 465 008	−0.16	.006	0.034	APCDD1L	Intron
	2	33 531 342	0.16	.006	0.036	RASGRP3	Intron
**Recent adult life events**							
	16	89 637 112	0.21	<.001	0.002	DPEP1	Intron
	12	10 213 513	0.2	.001	0.003	GABARAPL1	Intron
	16	57 565 171	0.17	.003	0.019	ADGRG5	Intron
	1	236 818 624	0.17	.003	0.020	MTR	Intron
	8	108 379 481	−0.17	.004	0.022	EMC2	Intergenic

These results were adjusted for age, sex, DI, and CPZ equivalent (missing CPZ values were treated as 0).

Abbreviations: BP, base pair; Chr, chromosome; CPZ, chlorpromazine; DI, duration of illness; DMPs, differentially methylated positions; FWER, family-wise error rate; K-PERS, Korea polyenvironmental risk score; *r*, correlation coefficient; TSS, transcription start site.

**Table 5:** Correlation Between the Methylation Level of DMRs and K-PERS-I

**Subdomains of** **the K-PERS-I**	**Chr**	**Start**	**End**	***r***	***P*-value**	**FWER**	**Gene**	**Genomic location distribution**
**Childhood adversity**								
	9	14 910 298	14 910 449	−0.19	<.001	0.002	FREM1	Promoter-TSS
	1	161 525 401	161 525 480	−0.17	.001	0.008	HSPA6	Exon
	1	24 911 705	24 911 820	0.17	.002	0.011	MIR6731	Intron
	5	148 827 207	148 827 706	0.16	.004	0.024	ADRB2	Exon
	8	78 083 944	78 084 046	−0.15	.004	0.026	PKIA	Intergenic
	8	101 051 561	101 051 716	−0.15	.005	0.027	FLJ42969	Promoter-TSS
	7	4 819 596	4 819 812	−0.15	.005	0.032	SNORD165	Intron
	7	64 885 013	64 885 131	−0.15	.005	0.033	ZNF273	Intergenic
	19	42 323 593	42 323 656	−0.15	.006	0.038	MEGF8	Exon
	3	129 558 354	129 558 566	0.15	.007	0.042	H1FOO	Exon
**Obstetric complications**								
	19	42 323 593	42 323 656	−0.18	.001	0.009	MEGF8	Exon
	2	232 386 690	232 386 776	−0.17	.002	0.013	ECEL1P2	Noncoding
	16	51 636 926	51 637 023	−0.17	.002	0.014	LINC01571	Intergenic
	2	157 597 089	157 597 210	−0.17	.003	0.016	ACVR1C	Intron
	2	20 669 686	20 671 939	0.17	.003	0.017	GDF7	Exon
**Recent adult life events**								
	7	2 019 380	2 019 479	0.21	<.001	0.002	SNORA114	Intron
	17	8 163 995	8 164 217	−0.18	.002	0.009	VAMP2	Intergenic
	4	148 261 416	148 261 491	0.18	.002	0.011	NR3C2	Intron
	5	16 617 849	16 617 986	−0.18	.002	0.011	RETREG1	Promoter-TSS
	8	140 578 652	140 578 739	0.18	.002	0.015	AGO2	Intron

These results were adjusted for age, sex, DI, and CPZ equivalent (missing CPZ values were treated as 0).

Abbreviations: Chr, chromosome; CPZ, chlorpromazine; DI, duration of illness; DMRs, differentially methylated regions; FWER, family-wise error rate; K-PERS, Korea polyenvironmental risk score; *r*, correlation coefficient; TSS, transcription start site.

### Correlation of DMR Methylation Levels with K-PERS-I

Eleven DMR-annotated genes showed a significant correlation with childhood adversity. The top 10 genes were *FREM1* (adjusted *P* = .002), *HSPA6* (adjusted *P* = .008), *MIR6731* (adjusted *P* = .011), *ADRB2* (adjusted *P* = .024), *PKIA* (adjusted *P* = .026), *FLJ42969* (adjusted *P* = .027), *SNORD165* (adjusted *P* = .032), *ZNF273* (adjusted *P* = .033), *MEGF8* (adjusted *P* = .038), and *H1FOO* (adjusted *P* = .042). Eighteen DMR-annotated genes were significantly correlated with obstetric complications. The top five genes were *MEGF8* (adjusted *P* = .009), *ECEL1P2* (adjusted *P* = .013), *LINC01571* (adjusted *P* = .014), *ACVR1C* (adjusted *P* = .016), and *GDF7* (adjusted *P* = .017). Thirty-six DMR-annotated genes showed significant associations with recent adult life events, of which the top five were *SNORA114* (adjusted *P* = .002), *VAMP2* (adjusted *P* = .009), *NR3C2* (adjusted *P* = .011), *RETREG1* (adjusted *P* = .011), and *AGO2* (adjusted *P* = .015) (Table 5). Results for other subdomains are provided in Table S2.

### Overlap of SZ-associated MWAS Genes and SZ-associated GWAS Genes

*NCOR2* was the only gene that overlapped with other MWAS studies.^17^^,^^26^^,^^55^  *ITIH3* was the only gene that overlapped with previous GWAS findings in European populations.

## Discussion

Several lines of research suggest that epigenetic mechanisms play a role in the pathophysiology of SZ. However, only a few studies have examined associations between altered DNA methylation levels and environmental factors in SZ. Using Methyl-Seq, we identified several psychosis-associated DMPs and DMRs, some of which were correlated with the K-PERS-I score.

We identified 1138 psychosis-associated DMPs annotated to 901 genes. The top 10 annotated genes were involved in chromatin organization, neuronal transport, cell–cell interactions, or clathrin-mediated endocytosis. Additionally, 1611 psychosis-associated DMRs annotated to 1447 genes were identified. Among the top 10 annotated genes, several were involved in regulating dopamine transporter levels and neuroinflammatory responses. See Supplementary Discussion for details.

Among the top 10 DMP-annotated genes associated with childhood adversity, *NRADDP* was the highest-ranking gene. Although *NRADDP* is a pseudogene and nonfunctional in humans, its mouse ortholog, *Nradd*, has been shown to induce apoptosis in neuroblastoma cell lines.^60^  *SOX8* is integral to neuronal development,^61^ particularly in astrocytic differentiation^62^ and ear neurogenesis.^63^  *PIK3CD* plays a vital role in immune responses, including β-cell development, proliferation, and function.^64^  *FZD10-AS1* is a long noncoding RNA (lncRNA) transcribed from the antisense strand adjacent to the *FZD10* gene, which encodes Frizzled Class Receptor 10, a component of the Wnt signaling pathway.^65^ Although no direct evidence links *FZD10-AS1* to neuronal function, childhood trauma, or psychosis, it may influence these processes through its relationships with *FZD10* and Wnt signaling. *SCN10A* plays a critical role in neuronal excitability and pain perception,^66^ but no direct evidence currently links it to childhood trauma or psychosis. *CTSB* has been implicated in oxidative stress in SZ.^67^  *LINC01983*, *LINC01605*, *FAM53B-AS1*, and *LINC02222* are all lncRNAs, and their functions in the brain remain undetermined. Notably, all of these genes are newly identified markers and do not overlap with findings from previous studies on childhood adversity using MWAS^12^^,^^32^^,^^68-70^ or candidate gene approaches.^71-74^ Nevertheless, given their roles in neuronal development, immune function, and oxidative stress, it is plausible that childhood trauma may become embedded in the methylation profiles of these genes through various mechanisms.

Among the top five DMP-annotated genes associated with obstetric complications, *PFKFB3* serves as a key regulator of glycolysis with distinct roles in placental function. In neurons, its tight regulation is critical for preventing oxidative stress and maintaining cellular integrity.^75^ In the placenta, appropriate *PFKFB3* activity supports angiogenesis and overall placental health, which are vital for favorable pregnancy outcomes.^76^  *NMNAT3* plays a crucial role in nicotinamide adenine dinucleotide^+^ (NAD^+^) biosynthesis, contributing to neuroprotection.^77^ Its function in maintaining NAD^+^ levels may also be important for placental physiology.^78^  *PAK2* is essential for proper myelination of nerves^79^ and neuronal migration during development.^80^ Loss of *Pak2* in mice is associated with fetal death due to multiple developmental defects.^81^ The *APCDD1L* gene encodes a protein that is homologous to *APCDD1*, a known inhibitor of the Wnt signaling pathway, which has been implicated in embryonic development and neural differentiation.^82^ This finding suggests that *APCDD1L* may play an important role in brain formation and neuronal specialization. *RASGRP3* has been linked to embryonic development, particularly under diabetic conditions.^83^ A recent study indicated that placental pathophysiology and birth asphyxia may influence brain development trajectories, particularly in male individuals, who are at greater risk of developing SZ.^84^ These findings represent the first report of associations between psychosis-associated DMPs and obstetric complications. Notably, the top five genes were implicated in placental function or embryonic development, suggesting that these DMPs could serve as DNA methylation biomarkers for insults associated with obstetric complications.

Among the top five DMP-annotated genes associated with recent adult life events, *DPEP1* has been implicated in the metabolism of glutathione, an important antioxidant.^85^ Altered *DPEP1* expression can affect glutathione levels and oxidative stress,^86^ potentially impairing the stress response. GABARAPL1 interacts with GABA_A_ receptors, facilitating their trafficking to the neuronal membrane.^87^ Exposure to oxidative stress, such as treatment with hydrogen peroxide (H_2_O_2_), upregulates *GABARAPL1* mRNA, and protein levels in rat C6 glioma cells.^88^  *ADGRG5*, also known as *GPR114*, belongs to the adhesion G protein-coupled receptor (aGPCR) subfamily, which plays a key role in neural development and cell–environment interactions.^89^ Disruptions in MTR activity can affect S-adenosylmethionine levels, potentially impacting neurotransmitter production.^90^ Elevated homocysteine levels, resulting from impaired MTR activity, have been associated with increased oxidative stress.^91^ EMC2 plays a key role in regulating GABA_A_ receptor biogenesis in the endoplasmic reticulum membrane.^92^ Considering the roles of these genes in oxidative stress, neuronal development, and neurotransmitter production, it can be inferred that adult stresses leave epigenetic imprints on their DNA methylation through diverse biological mechanisms.

With the exception of *HSPA6*, *ADRB2*, *PKIA*, and *MEGF8*, the top 10 childhood adversity-associated DMR-annotated genes have not been investigated for their involvement in neuronal function or childhood adversity. HSPA6, a member of the heat shock protein 70 (HSP70) family, is an inducible protein involved in cellular repair and stress responses, including those in neurons.^93^ ADRB2 modulates neurotransmitter release and neuronal excitability, influencing memory formation and synaptic plasticity.^94^ Additionally, ADRB2 signaling has been shown to affect immune cell functions, including the suppression of cytokine secretion from CD8^+^ T cells.^95^ Genetic variations in *ADRB2* may interact with childhood trauma and influence the risk of developing posttraumatic stress disorder.^96^  *PKIA* plays a significant role in neurons, affecting processes such as synaptic plasticity, learning, and memory.^97^  *MEGF8* is strongly expressed throughout the nervous system; its loss disrupts axon guidance in the peripheral nervous system in mice.^98^ In summary, these genes do not overlap with findings from previous MWAS on childhood adversity. Inconsistencies across studies may be due to differences in patient heterogeneity (psychosis vs SZ), methods for assessing DNA methylation (Methyl-Seq vs microarray), and approaches to measuring childhood adversity (interview vs scale rating).

With the exception of *MEGF8* and *GDF7*, the top five DMR-annotated genes associated with obstetric complications have not been studied in relation to neuronal function or obstetric complications. MEGF8 is known to regulate axon guidance,^98^ whereas GDF7 has potent sensory neuron-inducing activity in vitro.^99^ Among the top five DMR-annotated genes associated with recent adult life events, *VAMP2* is involved in promoting vesicle fusion and neurotransmitter release.^100^  *NR3C2* encodes the mineralocorticoid receptor, which is critical for neuronal function^101^ and stress regulation.^102^ Changes in its methylation status are among the most widely replicated epigenetic modifications associated with childhood trauma in both patients with psychosis and HCs.^103^ Additionally, hypermethylation of the *NR3C2-4* region has been detected in female patients with SZ relative to HCs.^104^  *AGO2* is involved in the cellular stress response by modulating RNA silencing and interacting with stress granules.^105^ These findings highlight possible epigenetic pathways through which life stressors may influence neurotransmitter release and stress regulation.

We observed four overlapping genes (*LINC02222*, *ZNF273*, *SUN1*, and *LSP1*) between the K-PERS-associated DMP- and DMR-annotated genes. *LINC02222* is a lncRNA, and there is increasing evidence to support the involvement of lncRNAs in SZ pathogenesis.^106^  *ZNF273* belongs to the zinc finger protein family, which plays a crucial role in brain development and has been implicated in various neurological disorders.^107^  *SUN1* is involved in neuronal migration in mice.^108^ Mutations in *SUN1* disrupt neuronal migration, which is linked to neurological and psychiatric disorders, including SZ.^109^  *LSP1* plays a key role in immune system regulation.^110^ Although the environmental factors varied, the importance of these shared genes requires further investigation and validation.

Taken together, we identified new psychosis-associated DMPs and DMRs. Some of the annotated genes were related to neurite outgrowth, clathrin-mediated endocytosis, actin filaments/cytoskeleton, and lncRNAs. In addition, five genes overlapped between the top 50 DMP- and DMR-annotated genes. For the correlation results, we also observed new methylation markers and annotated genes associated with childhood adversity. These genes were primarily implicated in neuronal development, neurotransmitter release, synaptic plasticity, immune response, and oxidative stress. For obstetric complications, most of the top five DMP-annotated genes were linked to placental function, embryonic development, or gestation. For recent adult life events, the top five DMP- and DMR-annotated genes were related to neurotransmitter production and release, oxidative stress, and stress regulation.

The present study had several limitations. First, as the diagnoses of patients were heterogeneous, caution is required in the interpretation of the results. Second, although Methyl-Seq offers several advantages—such as high resolution and unbiased genome-wide coverage compared with microarray analysis—it covers only approximately 3% of all CpG sites in the genome. Third, in the absence of gene expression data, it was not possible to assess the molecular impact of DNA methylation, limiting interpretation of the downstream implications of our findings. Fourth, our results—particularly those concerning environmental factors—require validation in independent cohorts. Finally, although we adjusted for covariates such as DI and CPZ equivalent dosage in the correlation analyses, the most rigorous approach would be to recruit antipsychotic-naïve patients experiencing their first episode of psychosis. Despite these caveats, the present study represents the first attempt to investigate the relationships between psychosis-associated DMPs and DMRs and polyenvironmental risk factors for SZ using the K-PERS-I.

In conclusion, we identified altered DNA methylation sites and their annotated genes that may contribute to the development of SZ and other psychotic disorders and could serve as biomarkers of psychosis. Moreover, we demonstrated how environmental factors can be biologically embedded in the DNA methylation profiles of specific genes in individuals with psychosis. Ultimately, establishing causal pathways between environmental risk factors and DNA methylation may lead to the discovery of novel therapeutic targets.

## Supplementary Material

Supplementary_materials_sgaf027
