# Supplementary material for: Impacts of Polyenvironmental Factors on DNA Methylation in Patients With Psychosis
Source: Schizophr Bull Open. 2025 Nov 3;6(1):sgaf027. doi: 10.1093/schizbullopen/sgaf027 (PMC12667615; doi:10.1093/schizbullopen/sgaf027)
Supplement: Supplementary_materials_sgaf027 [file supplementary_materials_sgaf027.docx]

**Supplementary discussion**

**1. Discussion on the psychosis-associated DMPs and DMRs**

For genomic distribution of DMPs and DMRs, most were located within introns, intergenic regions, and promoter-transcription start site (TSS) region. This finding indicates that altered DNA methylation in psychosis occurs not only in the promoter regions but also within gene bodies and intergenic region. Anastasiadi et al. ^1^ reported a consistent inverse correlation between DNA methylation of the first intron and gene expression across various tissues and species. However, the roles of DNA methylation in gene bodies and intergenic regions in relation to mental disorders remain largely unexplored.

For the top 10 DMP-annotated genes, specific studies in psychosis are scarce. The first ranked gene, *PLPP7*, encodes a nuclear envelope protein that plays a crucial role in maintaining nuclear integrity and chromatin organization, particularly in muscle cells ^2^. Its similar roles in neuronal contexts need to be investigated. *DNHD1* is implicated in sperm motility and cellular movement, but its direct role in neurological functions or SZ has not been explored. *HPS4* polymorphisms have been associated with variations in cognitive performance in SZ ^3^. ***ST3GAL4*** encodes the enzyme **ST3 beta-galactoside alpha-2,3-sialyltransferase 4**, which is involved in the sialylation of glycoproteins and glycolipids, processes crucial for cell-cell interactions and signaling ^4^. ***FCHSD2*** encodes a protein involved in clathrin-mediated endocytosis, a process essential for synaptic function and neuronal health ^5^. Disruptions in endocytic pathways have been implicated in neurodegenerative diseases ^6^, including SZ ^7^. Taken together, although there is a paucity of studies on the roles of these top 10 DMP-annotated genes in psychosis, altered methylation level of these DMPs may serve as novel biological markers for psychosis or SZ. Among the top 50 DMP-annotated genes, *ABLIM2*, *IQGAP3*, *SSH2* and *KAZN* are implicated in the regulation of actin filaments or actin cytoskeleton ^8-11^. Actin dysregulation has been proposed as a convergent mechanism of mental disorders ^12^ including SZ ^13^. *ELDR*, *VIM-AS1*, *LGALS8-AS1*, and *LINC00399* are long non-coding RNA (lncRNA). Emerging evidence indicates the involvement of lncRNA in SZ pathogenesis and highlights their potential as biomarkers and therapeutic targets ^14^.

For the top 10 DMR-annotated genes, direct evidence linking them to psychosis is limited, with the exception of *RGS12*. Exome sequencing in sporadic cases of SZ has identified de novo mutations in the *RGS12* gene ^15^. Rgs12-null mice exhibit increased dopamine transporter levels and enhanced dopamine uptake in the ventral striatum, leading to reduced hyperlocomotion in response to psychostimulants like amphetamine and cocaine ^16^. Given the expression of *GPR84* in microglia and its role in neuroinflammatory processes ^17^, it is plausible that dysregulation of *GPR84* may contribute to neuroinflammatory mechanisms implicated in SZ. Among the top 50 DMR-annotated genes, *LINC02470*, *LINC01100*, *LINC00670*, *LOC107984399*, *LINC02361*, *FOXCUT* and *LOC101927243* are classified as lncRNA or ncRNA. Although mRNA levels or protein expression levels are not directly related to DNA methylation levels, elevated antibody titers against *HSP70* ^18^, increased *FZD7* mRNA expression in blood ^19^, and decreased *LHX6* mRNA levels in the prefrontal cortex ^20^ have been observed in SZ. Further research is needed to elucidate the specific roles of DMR-annotated genes in the context of psychosis.

*NCOR2* was the only gene found to be overlapped between our top 50 DMP-annotated genes and significant DMP-annotated genes reported in previous blood-based MWAS in SZ. *NCOR2* functions as a key regulator of gene expression in neurons, influencing memory, and synaptic plasticity ^21^. Montano et al. ^22^ identified differential DNA methylation of *NCOR2* gene in individuals with SZ, indicating that epigenetic modifications of this gene may play a role in the disorder's pathogenesis. Interestingly, we identified five overlapping genes (*CTSB*, *DROSHA*, *FAM53A*, *PWP1*, and *RRN3P2*) between the top 50 DMP- and DMR-annotated genes. The **overlap between DMP- and DMR-annotated genes** suggests **stronger and more functionally relevant epigenetic regulation** compared to genes found in **only one category. This overlap** can be particularly valuable for prioritizing **candidate genes** for further investigation in psychosis studies. Especially, *CTSB* has been identified as one of the hub genes associated with oxidative stress in SZ ^23^. *DROSHA* involved in primary miRNA processing was found to be significantly upregulated in the dorsolateral prefrontal cortex in SZ ^24^. Lastly, we found only one overlapping gene (*ITIH3*) between our top 100 DMP- or DMR-annotated genes and significant SZ susceptibility genes measured by blood-based GWAS in both Asian and European populations. This finding contrasts with other MWAS that reported a higher degree of overlap with the GWAS in SZ ^25-28^.

**2. Discussion on the correlation results with other three factors of the K-PERS-I**

Among the top five DMP-annotated genes associated with paternal age at birth, *RBFOX3* plays a critical role in various neuronal processes such as neuronal differentiation ^29^ and neuronal circuitry balance ^30^. *KLHDC7A*, a member of the *KLHDC* subfamily, has been implicated in neurological functions ^31^. *DPH3P1* is classified as a pseudogene. *NMI* gene regulates cellular responses, promotes apoptosis, and suppresses tumor growth ^32^. The function of *UBALD2*, particularly in neuronal activity and its potential relation to paternal age at birth, remains unclear. Overall, the roles of these five genes in relation to advanced paternal age are largely underexplored. Although both animal and human studies indicate that advanced paternal age is associated with adverse offspring health outcomes ^33, 34^, no study has yet examined the association between DMPs and advanced paternal age in patients with SZ.

Among the top five DMP-annotated genes associated with pSES, *CTSB* is involved in neurite outgrowth ^35^ and memory functions ^36^. *ZNF273* belong to Zinc finger proteins family, which plays a crucial role in brain development and are implicated in various neurological disorders ^37^, including SZ ^38^. *RAET1E-AS1*, a lncRNA, has limited information regarding its specific role in neuronal function. *BRDT* is **primarily expressed in testis** and essential for **spermatogenesis.** It has not been directly investigated in relation to neurological functions or SZ. It may be speculated that pSES influences neurite outgrowth and brain development through DNA methylation in several genes found.

Among the top five DMP-annotated genes associated with urbanicity, *HPD* is an enzyme that plays a crucial role in the catabolic pathway of the amino acid tyrosine ^39^. While *HPD*'s primary expression is in hepatic and renal tissues, its role in tyrosine metabolism could indirectly impacts neurotransmitter synthesis. *TGFBI* is known to play a role in cell adhesion and extracellular matrix interactions, which are crucial for immune responses ^40^. However, specific immunological associations with SZ remain to be fully elucidated. Mutations in *FAM160B1* have been associated with microcephaly and severe intellectual disability ^41^, indicating its importance in nervous system development and function. *TECPR1* is highly expressed in the brain and involved in autophagy ^42^, a process implicated in various neurological disorders including SZ ^43^. RGS12 is involved in neuronal differentiation ^44^ and serotonergic neurotransmission ^45^. Overall, these findings may shed light on the mechanisms through which urbanicity leaves methylation signatures on certain genes.

Among the top DMR-annotated genes associated with paternal age at birth or pSES, all genes have not been investigated in relation to neuronal function and associated environmental factors. Among the top five DMR-annotated genes associated with urbanicity, the protocadherin beta (*PCDHB*) gene cluster, including *PCDHB4* and *PCDHB6*, is crucial for neural circuit and axon targeting ^46, 47^. *OLFM1*, also known as noelin 1 or pancortin, is abundantly expressed in the brain and is involved in neural development and axon growth ^48^. However, specific involvement of all five genes in processes related to urbanicity has not been established.

**References**

1. Anastasiadi D, Esteve-Codina A, Piferrer F. Consistent inverse correlation between DNA methylation of the first intron and gene expression across tissues and species. *Epigenetics Chromatin*. 2018;11(1):37.
2. Ramirez-Martinez A, Zhang Y, Chen K, et al. The nuclear envelope protein Net39 is essential for muscle nuclear integrity and chromatin organization. Nat Commun. 2021;12(1):690. doi:10.1038/s41467-021-20987-x
3. Kuratomi G, Saito A, Ozeki Y, et al. Association of the Hermansky-Pudlak syndrome type 4 (HPS4) gene variants with cognitive function in patients with schizophrenia and healthy subjects. BMC Psychiatry. 2013;13:276. doi:10.1186/1471-244X-13-276
4. Zhu W, Zhou Y, Guo L, Feng S. Biological function of sialic acid and sialylation in human health and disease. Cell Death Discov. 2024;10(1):415. doi:10.1038/s41420-024-02180-3
5. Almeida-Souza L, Frank RAW, García-Nafría J, et al. A Flat BAR Protein Promotes Actin Polymerization at the Base of Clathrin-Coated Pits. Cell. 2018;174(2):325-337.e14. <https://doi.org/10.1016/j.cell.2018.05.020>
6. Dong J, Tong W, Liu M, et al. Endosomal traffic disorders: a driving force behind neurodegenerative diseases. Transl Neurodegener. 2024;13(1):66. https://doi.org/10.1186/s40035-024-00460-7
7. Plooster M, Brennwald P, Gupton SL. Endosomal trafficking in schizophrenia. Curr Opin Neurobiol. 2022;74:102539. doi:10.1016/j.conb.2022.102539
8. Barrientos T, Frank D, Kuwahara K, et al. Two novel members of the ABLIM protein family, ABLIM-2 and -3, associate with STARS and directly bind F-actin. J Biol Chem. 2007;282(11):8393-8403. doi:10.1074/jbc.M607549200
9. Wang S, Watanabe T, Noritake J, et al. IQGAP3, a novel effector of Rac1 and Cdc42, regulates neurite outgrowth. J Cell Sci. 2007;120(Pt 4):567-577. doi:10.1242/jcs.03356
10. Chang J, Chen C, Li W, Abumaria N. TRPM7 Kinase Domain is Part of the Rac1-SSH2-cofilin Complex Regulating F-actin in the Mouse Nervous System. Neurosci Bull. 2023;39(6):989-993. doi:10.1007/s12264-023-01045-6
11. Sevilla LM, Nachat R, Groot KR, Watt FM. Kazrin regulates keratinocyte cytoskeletal networks, intercellular junctions and differentiation. J Cell Sci. 2008;121(Pt 21):3561-3569. doi:10.1242/jcs.029538
12. Yan Z, Kim E, Datta D, Lewis DA, Soderling SH. Synaptic Actin Dysregulation, a Convergent Mechanism of Mental Disorders?. J Neurosci. 2016;36(45):11411-11417. doi:10.1523/JNEUROSCI.2360-16.2016
13. Loe-Mie Y, Plançon C, Dubertret C, et al. De Novo Variants Found in Three Distinct Schizophrenia Populations Hit a Common Core Gene Network Related to Microtubule and Actin Cytoskeleton Gene Ontology Classes. Life (Basel). 2024;14(2):244. Published 2024 Feb 9. doi:10.3390/life14020244
14. Wu G, Du X, Li Z, et al. The emerging role of long non-coding RNAs in schizophrenia. Front Psychiatry. 2022;13:995956. doi:10.3389/fpsyt.2022.995956
15. Guipponi M, Santoni FA, Setola V, et al. Correction: Exome Sequencing in 53 Sporadic Cases of Schizophrenia Identifies 18 Putative Candidate Genes. PLoS One. 2015;10(10):e0141630. doi:10.1371/journal.pone.0141630
16. Gross JD, Kaski SW, Schroer AB, Wix KA, Siderovski DP, Setola V. Regulator of G protein signaling-12 modulates the dopamine transporter in ventral striatum and locomotor responses to psychostimulants. J Psychopharmacol. 2018;32(2):191-203. doi:10.1177/0269881117742100
17. Bouchard C, Pagé J, Bédard A, Tremblay P, Vallières L. G protein-coupled receptor 84, a microglia-associated protein expressed in neuroinflammatory conditions. Glia. 2007;55(8):790-800. doi:10.1002/glia.20506
18. Schwarz MJ, Riedel M, Gruber R, Ackenheil M, Müller N. Antibodies to heat shock proteins in schizophrenic patients: implications for the mechanism of the disease. Am J Psychiatry. 1999;156(7):1103-1104. doi:10.1176/ajp.156.7.1103
19. Hoseth EZ, Krull F, Dieset I, et al. Exploring the Wnt signaling pathway in schizophrenia and bipolar disorder. Transl Psychiatry. 2018;8(1):55. doi:10.1038/s41398-018-0102-1
20. Volk DW, Matsubara T, Li S, et al. Deficits in transcriptional regulators of cortical parvalbumin neurons in schizophrenia. Am J Psychiatry. 2012;169(10):1082-1091. doi:10.1176/appi.ajp.2012.12030305
21. Zhou W, He Y, Rehman AU, et al. Loss of function of NCOR1 and NCOR2 impairs memory through a novel GABAergic hypothalamus-CA3 projection [published correction appears in Nat Neurosci. 2019 Sep;22(9):1533. doi: 10.1038/s41593-019-0449-5.]. Nat Neurosci. 2019;22(2):205-217. doi:10.1038/s41593-018-0311-1
22. Montano C, Taub MA, Jaffe A, et al. Association of DNA Methylation Differences With Schizophrenia in an Epigenome-Wide Association Study. JAMA Psychiatry. 2016;73(5):506-514. doi:10.1001/jamapsychiatry.2016.0144
23. Zhu XM, Chen J, Ba HJ, et al. Revealing the Oxidative Stress-Related Molecular Characteristics and Potential Therapeutic Targets of Schizophrenia through Integrated Gene Expression Data Analysis. Mol Neurobiol. Published online April 11, 2025. doi:10.1007/s12035-025-04924-3
24. Beveridge NJ, Gardiner E, Carroll AP, Tooney PA, Cairns MJ. Schizophrenia is associated with an increase in cortical microRNA biogenesis. Mol Psychiatry. 2010;15(12):1176-1189. doi:10.1038/mp.2009.84
25. Hannon E, Dempster EL, Mansell G, et al. DNA methylation meta-analysis reveals cellular alterations in psychosis and markers of treatment-resistant schizophrenia. Elife. 2021;10:e58430. doi:10.7554/eLife.58430
26. Hannon E, Dempster E, Viana J, et al. An integrated genetic-epigenetic analysis of schizophrenia: evidence for co-localization of genetic associations and differential DNA methylation. Genome Biol. 2016;17(1):176. doi:10.1186/s13059-016-1041-x
27. Liu Y, Lang B, Smith R, C et al. Methylomic Alteration in Peripheral Blood Lymphocytes of Prodromal Stage and First-Episode Chinese Han Schizophrenia Patients. SSRN Electronic Journal. 2021.430160; <https://doi.org/10.2139/ssrn.3844823>
28. Shen L, Lv X, Huang H, et al. Genome-wide analysis of DNA methylation in 106 schizophrenia family trios in Han Chinese. EBioMedicine. 2021;72:103609. doi:10.1016/j.ebiom.2021.103609
29. Kim KK, Nam J, Mukouyama YS, Kawamoto S. Rbfox3-regulated alternative splicing of Numb promotes neuronal differentiation during development. J Cell Biol. 2013;200(4):443-458. doi:10.1083/jcb.201206146
30. Lin YS, Kuo KT, Chen SK, Huang HS. RBFOX3/NeuN is dispensable for visual function. PLoS One. 2018;13(2):e0192355. Published 2018 Feb 5. doi:10.1371/journal.pone.0192355
31. Pilcher C, Buco PAV, Truong JQ, et al. Characteristics of the Kelch domain containing (KLHDC) subfamily and relationships with diseases. FEBS Lett. Published online January 30, 2025. doi:10.1002/1873-3468.15108
32. Park Y, Guan X, Han SJ. N-Myc and STAT Interactor is an Endometriosis Suppressor. Int J Mol Sci. 2024;25(15):8145. Published 2024 Jul 26. doi:10.3390/ijms25158145
33. Denomme MM, McCallie BR, Haywood ME, Parks JC, Schoolcraft WB, Katz-Jaffe MG. Paternal aging impacts expression and epigenetic markers as early as the first embryonic tissue lineage differentiation. Hum Genomics. 2024;18(1):32. Published 2024 Mar 26. doi:10.1186/s40246-024-00599-4
34. Xu X, Miao Z, Sun M, Wan B. Epigenetic Mechanisms of Paternal Stress in Offspring Development and Diseases. Int J Genomics. 2021;2021:6632719. Published 2021 Jan 19. doi:10.1155/2021/6632719
35. Jiang M, Meng J, Zeng F, et al. Cathepsin B inhibition blocks neurite outgrowth in cultured neurons by regulating lysosomal trafficking and remodeling. J Neurochem. 2020;155(3):300-312. doi:10.1111/jnc.15032
36. Niemeyer C, Matosin N, Kaul D, Philipsen A, Gassen NC. The Role of Cathepsins in Memory Functions and the Pathophysiology of Psychiatric Disorders. Front Psychiatry. 2020;11:718. doi:10.3389/fpsyt.2020.00718
37. Bu S, Lv Y, Liu Y, Qiao S, Wang H. Zinc Finger Proteins in Neuro-Related Diseases Progression. Front Neurosci. 2021;15:760567. doi:10.3389/fnins.2021.760567
38. Dong F, Mao J, Chen M, Yoon J, Mao Y. Schizophrenia risk ZNF804A interacts with its associated proteins to modulate dendritic morphology and synaptic development. Mol Brain. 2021;14(1):12. doi:10.1186/s13041-021-00729-2
39. Neve S, Aarenstrup L, Tornehave D, et al. Tissue distribution, intracellular localization and proteolytic processing of rat 4-hydroxyphenylpyruvate dioxygenase. Cell Biol Int. 2003;27(8):611-624. doi:10.1016/s1065-6995(03)00117-3
40. Chen Y, Zhao H, Feng Y, et al. Pan-Cancer Analysis of the Associations of TGFBI Expression With Prognosis and Immune Characteristics. Front Mol Biosci. 2021;8:745649. doi:10.3389/fmolb.2021.745649
41. Mavioğlu RN, Kara B, Akansel G, Nalbant G, Tolun A. FAM160B1 deficit associated with microcephaly, severe intellectual disability, ataxia, behavioral abnormalities and speech problems. Clin Genet. 2019;96(5):456-460. doi:10.1111/cge.13612
42. Kim JH, Hong SB, Lee JK, et al. Insights into autophagosome maturation revealed by the structures of ATG5 with its interacting partners. Autophagy. 2015;11(1):75-87. doi:10.4161/15548627.2014.984276
43. Schneider JL, Miller AM, Woesner ME. Autophagy and Schizophrenia: A Closer Look at How Dysregulation of Neuronal Cell Homeostasis Influences the Pathogenesis of Schizophrenia. Einstein J Biol Med. 2016;31(1-2):34-39. doi:10.23861/EJBM201631752
44. Willard MD, Willard FS, Li X, Cappell SD, Snider WD, Siderovski DP. Selective role for RGS12 as a Ras/Raf/MEK scaffold in nerve growth factor-mediated differentiation. EMBO J. 2007;26(8):2029-2040. doi:10.1038/sj.emboj.7601659
45. White AN, Gross JD, Kaski SW, et al. Genetic deletion of Rgs12 in mice affects serotonin transporter expression and function in vivo and ex vivo. J Psychopharmacol. 2020;34(12):1393-1407. doi:10.1177/0269881120944160
46. Hasegawa S, Kobayashi H, Kumagai M, et al. Clustered Protocadherins Are Required for Building Functional Neural Circuits. Front Mol Neurosci. 2017;10:114. Published 2017 Apr 24. doi:10.3389/fnmol.2017.00114
47. Hasegawa S, Kumagai M, Hagihara M, et al. Distinct and Cooperative Functions for the Protocadherin-α, -β and -γ Clusters in Neuronal Survival and Axon Targeting. Front Mol Neurosci. 2016;9:155. doi:10.3389/fnmol.2016.00155
48. Nakaya N, Sultana A, Lee HS, Tomarev SI. Olfactomedin 1 interacts with the Nogo A receptor complex to regulate axon growth. J Biol Chem. 2012;287(44):37171-37184. doi:10.1074/jbc.M112.389916

**Material 1.** Definition of subdomains of the K-PERS-I and its scoring

Paternal age at birth equal to or greater than 36 years old was scored as 0.5, and under 36 years old, as -0.5. Obstetric complications were identified based on whether the subject was born with a low birth weight (less than 2.5 kg) (2 for yes and 0 for no). Parental socioeconomic status during childhood (up to 12 years old) was classified as difficult (1) or good (0) based on parents’ education and occupation. Urbanicity was considered present (0) when a person was raised in a city for more than 50% of childhood (up to 12 years old). Rural living was given a score of 3. Childhood adversity encompasses emotional abuse, emotional neglect, physical abuse, and sexual abuse. If at least one trauma was present, it was scored as 4.5; otherwise, as 0. Adult life events evaluated whether ones experienced at least two adverse events (5.5 for yes and -2 for no) among living alone, financial hardship, and difficulties in social relationships and occupational or academic functioning at least 6 months prior to the development of psychotic symptoms (Jeon et al., 2022).

**Table S1.** **Correlation between the methylation level of DMPs and K-PERS-I**

| **Subdomains of**  **the K-PERS-I** | **Chr** | **Position (bp)** | **r** | **p-value** | **FWER** | **Gene** | **Genomic location distribution** |
| --- | --- | --- | --- | --- | --- | --- | --- |
| **Childhood adversity** |  |  |  |  |  |  |  |
|  | 3 | 47001749 | 0.19 | <0.001 | 0.002 | NRADDP | Exon |
|  | 8 | 37517941 | -0.18 | 0.001 | 0.005 | LINC01605 | Intron |
|  | 16 | 1020029 | 0.18 | 0.001 | 0.005 | SOX8 | Intergenic |
|  | 1 | 9734248 | 0.17 | 0.001 | 0.009 | PIK3CD | Intron |
|  | 12 | 130138965 | 0.16 | 0.004 | 0.022 | FZD10-AS1 | Intergenic |
|  | 5 | 180684145 | 0.15 | 0.005 | 0.028 | LINC02222 | Promoter-TSS |
|  | 3 | 38823447 | 0.15 | 0.005 | 0.032 | SCN10A | Intergenic |
|  | 8 | 11915156 | -0.15 | 0.006 | 0.038 | CTSB | Intergenic |
|  | 10 | 124626167 | 0.15 | 0.006 | 0.039 | FAM53B-AS1 | Intron |
|  | 3 | 195860229 | 0.15 | 0.007 | 0.043 | LINC01983 | Intron |
|  | 8 | 141151823 | 0.14 | 0.008 | 0.046 | DENND3 | Exon |
|  | 14 | 78566162 | -0.14 | 0.008 | 0.048 | NRXN3 | Intron |
| **Obstetric complications** |  |  |  |  |  |  |  |
|  | 10 | 6178247 | -0.18 | 0.002 | 0.009 | PFKFB3 | Intron |
|  | 3 | 139679060 | 0.16 | 0.004 | 0.026 | NMNAT3 | Intergenic |
|  | 3 | 196738942 | 0.16 | 0.005 | 0.033 | PAK2 | Promoter-TSS |
|  | 20 | 58465008 | -0.16 | 0.006 | 0.034 | APCDD1L | Intron |
|  | 2 | 33531342 | 0.16 | 0.006 | 0.036 | RASGRP3 | Intron |
|  | 5 | 6707869 | -0.15 | 0.008 | 0.045 | LINC02102 | Promoter-TSS |
| **Paternal age at birth** |  |  |  |  |  |  |  |
|  | 17 | 79393675 | 0.18 | 0.001 | 0.005 | RBFOX3 | Intron |
|  | 1 | 18441298 | -0.17 | 0.001 | 0.009 | KLHDC7A | Intergenic |
|  | 20 | 62831177 | 0.16 | 0.003 | 0.018 | DPH3P1 | Intron |
|  | 2 | 151297493 | -0.16 | 0.003 | 0.021 | NMI | Intergenic |
|  | 20 | 62831114 | 0.15 | 0.004 | 0.023 | DPH3P1 | Intron |
|  | 17 | 76274077 | 0.15 | 0.005 | 0.028 | UBALD2 | TTS |
|  | 2 | 37243614 | -0.15 | 0.005 | 0.028 | NDUFAF7 | Intron |
|  | 17 | 76274099 | 0.15 | 0.006 | 0.034 | UBALD2 | TTS |
|  | 6 | 44272457 | -0.15 | 0.006 | 0.035 | TMEM151B | Intron |
|  | 5 | 181233341 | 0.15 | 0.006 | 0.035 | SNORD96A | Intron |
|  | 7 | 1120821 | -0.14 | 0.007 | 0.043 | C7orf50 | Intron |
|  | 7 | 1124390 | -0.14 | 0.007 | 0.045 | C7orf50 | Intron |
|  | 6 | 44284892 | -0.14 | 0.008 | 0.046 | TCTE1 | Intron |
|  | 17 | 76274101 | 0.14 | 0.008 | 0.049 | UBALD2 | TTS |
| **Parental SES** |  |  |  |  |  |  |  |
|  | 8 | 11862855 | 0.15 | 0.004 | 0.024 | CTSB | Intron |
|  | 14 | 24396928 | 0.15 | 0.004 | 0.024 | NYNRIN | Intergenic |
|  | 8 | 11862367 | 0.15 | 0.004 | 0.025 | CTSB | Intron |
|  | 7 | 64881766 | 0.15 | 0.004 | 0.025 | ZNF273 | Intergenic |
|  | 6 | 149846263 | -0.15 | 0.005 | 0.027 | RAET1E-AS1 | Intron |
|  | 1 | 91952442 | -0.15 | 0.005 | 0.027 | BRDT | Intron |
|  | 17 | 32991812 | -0.15 | 0.005 | 0.029 | SPACA3 | Promoter-TSS |
|  | 3 | 32503450 | 0.15 | 0.005 | 0.029 | CMTM6 | Promoter-TSS |
|  | 10 | 121682644 | 0.15 | 0.005 | 0.030 | FGFR2 | Intergenic |
|  | 8 | 141151823 | 0.15 | 0.005 | 0.030 | DENND3 | Exon |
|  | 3 | 42224143 | -0.15 | 0.005 | 0.032 | CCK | Non-coding |
|  | 8 | 11862710 | 0.15 | 0.005 | 0.032 | CTSB | Intron |
|  | 19 | 1451256 | -0.15 | 0.006 | 0.033 | APC2 | Intron |
|  | 7 | 155573934 | 0.15 | 0.006 | 0.038 | CNPY1 | Intergenic |
|  | 7 | 135795681 | -0.15 | 0.007 | 0.039 | FAM180A | Intergenic |
|  | 4 | 55634196 | 0.14 | 0.008 | 0.049 | NMU | Intron |
| **Recent adult life events** |  |  |  |  |  |  |  |
|  | 16 | 89637112 | 0.21 | <0.001 | 0.002 | DPEP1 | Intron |
|  | 12 | 10213513 | 0.2 | 0.001 | 0.003 | GABARAPL1 | Intron |
|  | 12 | 10213494 | 0.2 | <0.001 | 0.003 | GABARAPL1 | Intron |
|  | 16 | 57565171 | 0.17 | 0.003 | 0.019 | ADGRG5 | Intron |
|  | 1 | 236818624 | 0.17 | 0.003 | 0.020 | MTR | Intron |
|  | 8 | 108379481 | -0.17 | 0.004 | 0.022 | EMC2 | Intergenic |
|  | 7 | 64192343 | -0.16 | 0.005 | 0.029 | ZNF735 | Intergenic |
|  | 14 | 92598642 | -0.16 | 0.005 | 0.030 | RIN3 | Intron |
|  | 18 | 58124212 | -0.16 | 0.007 | 0.040 | NEDD4L | Intron |
|  | 12 | 120639706 | -0.16 | 0.007 | 0.040 | CABP1 | Promoter-TSS |
|  | 16 | 57722732 | -0.16 | 0.007 | 0.042 | KATNB1 | Exon |
| **Urbanicity** |  |  |  |  |  |  |  |
|  | 12 | 121858879 | 0.23 | <0.001 | <0.001 | HPD | Promoter-TSS |
|  | 5 | 136045658 | -0.2 | <0.001 | 0.001 | TGFBI | Intron |
|  | 10 | 114876564 | -0.2 | <0.001 | 0.001 | FAM160B1 | Intron |
|  | 7 | 98238722 | -0.19 | <0.001 | 0.003 | TECPR1 | Intron |
|  | 4 | 3293846 | -0.17 | 0.001 | 0.008 | RGS12 | Intergenic |
|  | 4 | 3293828 | -0.17 | 0.001 | 0.008 | RGS12 | Intergenic |
|  | 1 | 39526110 | 0.17 | 0.002 | 0.011 | OXCT2P1 | 3' UTR |
|  | 4 | 175907375 | 0.16 | 0.002 | 0.015 | GPM6A | Intron |
|  | 1 | 39526134 | 0.16 | 0.003 | 0.016 | OXCT2P1 | 3' UTR |
|  | 4 | 3317827 | 0.15 | 0.004 | 0.025 | RGS12 | Exon |
|  | 4 | 94584825 | -0.15 | 0.005 | 0.031 | PDLIM5 | Intron |
|  | 16 | 70156499 | 0.15 | 0.006 | 0.036 | LOC400541 | Exon |
|  | 3 | 8662809 | 0.15 | 0.006 | 0.037 | SSUH2 | Intergenic |
|  | 4 | 3373249 | -0.15 | 0.006 | 0.039 | RGS12 | Intron |
|  | 4 | 3373251 | -0.14 | 0.007 | 0.042 | RGS12 | Intron |
|  | 2 | 178807688 | -0.14 | 0.008 | 0.046 | TTN | Promoter-TSS |
|  | 4 | 3293814 | -0.14 | 0.008 | 0.048 | RGS12 | Intergenic |
| **Total score** |  |  |  |  |  |  |  |
|  | 8 | 118108171 | -0.21 | 0.001 |  | EXT1 | Intron |
|  | 16 | 1020029 | 0.19 | 0.002 |  | SOX8 | Intergenic |
|  | 1 | 236818624 | 0.17 | 0.005 |  | MTR | Intron |
|  | 9 | 34371018 | 0.17 | 0.005 |  | MYORG | Exon |
|  | 1 | 223700194 | -0.17 | 0.007 |  | CAPN2 | Intergenic |
|  | 10 | 3239255 | 0.17 | 0.007 |  | PITRM1 | Intergenic |
|  | 7 | 5987310 | 0.17 | 0.007 |  | RSPH10B2 | Exon |
|  | 17 | 76274088 | 0.17 | 0.008 |  | UBALD2 | TTS |
|  | 7 | 1157030 | -0.17 | 0.008 |  | ZFAND2A | Intron |
|  | 8 | 11915156 | -0.16 | 0.010 |  | CTSB | Intergenic |
|  | 17 | 76274077 | 0.16 | 0.011 |  | UBALD2 | TTS |
|  | 11 | 3232802 | -0.16 | 0.012 |  | MRGPRE | Promoter-TSS |
|  | 7 | 1106180 | -0.16 | 0.012 |  | GPER1 | Intron |
|  | 2 | 42770798 | 0.16 | 0.012 |  | OXER1 | Intron |
|  | 18 | 80078363 | -0.16 | 0.013 |  | RBFADN | Intron |
|  | 4 | 94584825 | -0.16 | 0.013 |  | PDLIM5 | Intron |
|  | 7 | 995204 | -0.16 | 0.013 |  | CYP2W1 | Intron |
|  | 9 | 34371015 | 0.16 | 0.013 |  | MYORG | Exon |
|  | 1 | 3218420 | 0.15 | 0.014 |  | MIR4251 | Intron |
|  | 9 | 34371012 | 0.15 | 0.014 |  | MYORG | Exon |
|  | 17 | 76274099 | 0.15 | 0.015 |  | UBALD2 | TTS |
|  | 17 | 76274101 | 0.15 | 0.015 |  | UBALD2 | TTS |
|  | 2 | 178807688 | -0.15 | 0.015 |  | TTN | Promoter-TSS |
|  | 2 | 3627566 | -0.15 | 0.016 |  | COLEC11 | Intron |
|  | 1 | 9734248 | 0.15 | 0.016 |  | PIK3CD | Intron |
|  | 7 | 1120821 | -0.15 | 0.017 |  | C7orf50 | Intron |
|  | 17 | 76274036 | 0.15 | 0.018 |  | UBALD2 | TTS |
|  | 2 | 24843118 | -0.15 | 0.019 |  | CENPO | Intron |
|  | 5 | 31470784 | 0.15 | 0.019 |  | DROSHA | Intron |
|  | 14 | 24396928 | 0.15 | 0.019 |  | NYNRIN | Intergenic |
|  | 7 | 151023339 | 0.15 | 0.020 |  | ATG9B | Intron |
|  | 5 | 31470750 | 0.14 | 0.022 |  | DROSHA | Intron |
|  | 3 | 197019212 | -0.14 | 0.023 |  | MELTF | 3' UTR |
|  | 17 | 3014055 | 0.14 | 0.023 |  | LOC101927911 | Intron |
|  | 4 | 3293846 | -0.14 | 0.024 |  | RGS12 | Intergenic |
|  | 1 | 93871380 | 0.14 | 0.024 |  | DNTTIP2 | Intron |
|  | 11 | 128889560 | 0.14 | 0.024 |  | KCNJ5 | Intergenic |
|  | 20 | 58465008 | -0.14 | 0.024 |  | APCDD1L | Intron |
|  | 4 | 146174479 | -0.14 | 0.026 |  | LSM6 | Intergenic |
|  | 3 | 33046638 | -0.14 | 0.026 |  | GLB1 | Intron |
|  | 2 | 37243614 | -0.14 | 0.026 |  | NDUFAF7 | Intron |
|  | 19 | 1883435 | -0.14 | 0.026 |  | ABHD17A | Intron |
|  | 13 | 113024033 | -0.14 | 0.026 |  | MCF2L | Intron |
|  | 13 | 98432429 | 0.14 | 0.026 |  | STK24 | Intron |
|  | 22 | 23306292 | -0.14 | 0.027 |  | LINC02556 | Intron |
|  | 7 | 129205862 | -0.14 | 0.028 |  | SMO | Intron |
|  | 22 | 22791613 | 0.14 | 0.028 |  | MIR650 | Intergenic |
|  | 1 | 181088144 | 0.14 | 0.029 |  | IER5 | Promoter-TSS |
|  | 10 | 127176358 | -0.14 | 0.029 |  | INSYN2A | Exon |
|  | 10 | 124626167 | 0.14 | 0.029 |  | FAM53B-AS1 | Intron |
|  | 7 | 98238722 | -0.14 | 0.030 |  | TECPR1 | Intron |
|  | 4 | 3293828 | -0.13 | 0.034 |  | RGS12 | Intergenic |
|  | 2 | 23531956 | -0.13 | 0.038 |  | KLHL29 | Intron |
|  | 13 | 37175660 | -0.13 | 0.038 |  | CSNK1A1L | Intergenic |
|  | 6 | 149866738 | -0.13 | 0.038 |  | LRP11 | Intron |
|  | 5 | 31470795 | 0.13 | 0.040 |  | DROSHA | Intron |
|  | 18 | 2909834 | 0.13 | 0.041 |  | LOC727896 | Intron |
|  | 16 | 86370887 | 0.13 | 0.041 |  | LINC00917 | Intergenic |
|  | 3 | 195860229 | 0.13 | 0.042 |  | LINC01983 | Intron |
|  | 7 | 779588 | -0.13 | 0.042 |  | SUN1 | Intron |
|  | 5 | 1818014 | -0.13 | 0.044 |  | NDUFS6 | Intergenic |
|  | 6 | 11714330 | -0.13 | 0.044 |  | ADTRP | 3' UTR |
|  | 17 | 78874378 | -0.12 | 0.047 |  | CEP295NL | Intron |
|  | 11 | 1849953 | 0.12 | 0.047 |  | LSP1 | Intergenic |
|  | 10 | 11870468 | 0.12 | 0.047 |  | PROSER2-AS1 | 3' UTR |
|  | 16 | 70156499 | 0.12 | 0.048 |  | LOC400541 | Exon |
|  | 1 | 225824577 | -0.12 | 0.049 |  | EPHX1 | Promoter-TSS |
|  | 2 | 24832762 | -0.12 | 0.049 |  | CENPO | Intron |
|  | 6 | 149889588 | -0.12 | 0.049 |  | RAET1E-AS1 | Promoter-TSS |
|  | 19 | 15021466 | 0.12 | 0.049 |  | CCDC105 | Exon |

These results were adjusted for age, sex, DI, and CPZ equivalent (missing CPZ values were treated as 0).

Note: BP: Base Pair; Chr: Chromosome; CPZ: Chlorpromazine; DI: Duration of illness; DMPs: Differentially Methylated Positions; FWER: Family-wise Error Rate; K-PERS: Korea Polyenvironmental Risk Score; r: Correlation Coefficient; SES: Socio-economic Status; TSS: Transcription Start Site.

**Table S2. Correlation between the methylation level of DMRs and K-PERS-I**

| **Subdomains of**  **the K-PERS-I** | **Chr** | **Start** | **End** | **r** | **p-value** | **FWER** | **Gene** | **Genomic location distribution** |
| --- | --- | --- | --- | --- | --- | --- | --- | --- |
| **Childhood adversity** |  |  |  |  |  |  |  |  |
|  | 9 | 14910298 | 14910449 | -0.19 | <0.001 | 0.002 | FREM1 | Promoter-TSS |
|  | 1 | 161525401 | 161525480 | -0.17 | 0.001 | 0.008 | HSPA6 | Exon |
|  | 1 | 24911705 | 24911820 | 0.17 | 0.002 | 0.011 | MIR6731 | Intron |
|  | 5 | 148827207 | 148827706 | 0.16 | 0.004 | 0.024 | ADRB2 | Exon |
|  | 8 | 78083944 | 78084046 | -0.15 | 0.004 | 0.026 | PKIA | Intergenic |
|  | 8 | 101051561 | 101051716 | -0.15 | 0.005 | 0.027 | FLJ42969 | Promoter-TSS |
|  | 7 | 4819596 | 4819812 | -0.15 | 0.005 | 0.032 | SNORD165 | Intron |
|  | 7 | 64885013 | 64885131 | -0.15 | 0.005 | 0.033 | ZNF273 | Intergenic |
|  | 19 | 42323593 | 42323656 | -0.15 | 0.006 | 0.038 | MEGF8 | Exon |
|  | 3 | 129558354 | 129558566 | 0.15 | 0.007 | 0.042 | H1FOO | Exon |
|  | 2 | 74415877 | 74416087 | -0.14 | 0.008 | 0.048 | C2orf81 | 5' UTR |
| **Obstetric complications** |  |  |  |  |  |  |  |  |
|  | 19 | 42323593 | 42323656 | -0.18 | 0.001 | 0.009 | MEGF8 | Exon |
|  | 2 | 232386690 | 232386776 | -0.17 | 0.002 | 0.013 | ECEL1P2 | Non-coding |
|  | 16 | 51636926 | 51637023 | -0.17 | 0.002 | 0.014 | LINC01571 | Intergenic |
|  | 2 | 157597089 | 157597210 | -0.17 | 0.003 | 0.016 | ACVR1C | Intron |
|  | 2 | 20669686 | 20671939 | 0.17 | 0.003 | 0.017 | GDF7 | Exon |
|  | 10 | 329263 | 329892 | -0.17 | 0.003 | 0.017 | ZMYND11 | Exon |
|  | 19 | 35433988 | 35434566 | -0.17 | 0.003 | 0.020 | FFAR2 | Intergenic |
|  | 9 | 19934355 | 19934405 | -0.16 | 0.004 | 0.024 | SLC24A2 | Intergenic |
|  | 18 | 70429936 | 70430210 | -0.16 | 0.006 | 0.033 | LINC01910 | Intergenic |
|  | 1 | 31788086 | 31788258 | -0.16 | 0.006 | 0.034 | ADGRB2 | Intergenic |
|  | 21 | 32393663 | 32393800 | -0.16 | 0.006 | 0.036 | URB1-AS1 | Promoter-TSS |
|  | 3 | 53666005 | 53666345 | -0.16 | 0.006 | 0.037 | CACNA1D | Intron |
|  | 7 | 64885013 | 64885131 | -0.15 | 0.007 | 0.041 | ZNF273 | Intergenic |
|  | 15 | 79970709 | 79970808 | 0.15 | 0.007 | 0.041 | BCL2A1 | Exon |
|  | 13 | 78659372 | 78660185 | -0.15 | 0.007 | 0.045 | OBI1 | Promoter-TSS |
|  | 3 | 125108043 | 125108176 | 0.15 | 0.008 | 0.046 | MIR5092 | Exon |
|  | 7 | 91557139 | 91557230 | 0.15 | 0.008 | 0.048 | FZD1 | Intergenic |
|  | 7 | 151805218 | 151805306 | -0.15 | 0.008 | 0.049 | LOC644090 | Intron |
| **Paternal age at birth** |  |  |  |  |  |  |  |  |
|  | 19 | 48196555 | 48196695 | 0.16 | 0.003 | 0.017 | ZSWIM9 | Exon |
|  | 2 | 117858496 | 117859180 | 0.14 | 0.008 | 0.047 | LOC107985939 | Intergenic |
| **Parental SES** |  |  |  |  |  |  |  |  |
|  | 13 | 20142797 | 20143161 | 0.17 | 0.001 | 0.009 | GJA3 | Exon |
|  | 1 | 10429969 | 10430155 | 0.16 | 0.002 | 0.014 | CENPS-CORT | Promoter-TSS |
|  | 16 | 87701142 | 87701262 | -0.15 | 0.005 | 0.027 | KLHDC4 | Intergenic |
| **Recent adult life events** |  |  |  |  |  |  |  |  |
|  | 7 | 2019380 | 2019479 | 0.21 | <0.001 | 0.002 | SNORA114 | Intron |
|  | 17 | 8163995 | 8164217 | -0.18 | 0.002 | 0.009 | VAMP2 | Intergenic |
|  | 4 | 148261416 | 148261491 | 0.18 | 0.002 | 0.011 | NR3C2 | Intron |
|  | 5 | 16617849 | 16617986 | -0.18 | 0.002 | 0.011 | RETREG1 | Promoter-TSS |
|  | 8 | 140578652 | 140578739 | 0.18 | 0.002 | 0.015 | AGO2 | Intron |
|  | 12 | 54369346 | 54369585 | 0.17 | 0.003 | 0.016 | GPR84 | 3' UTR |
|  | 19 | 38214294 | 38214371 | -0.17 | 0.003 | 0.017 | DPF1 | Intron |
|  | 22 | 19892598 | 19892702 | -0.17 | 0.003 | 0.019 | RTL10 | Intron |
|  | 2 | 224024328 | 224024418 | -0.17 | 0.004 | 0.025 | SERPINE2 | Intron |
|  | 22 | 46039238 | 46039260 | 0.17 | 0.004 | 0.025 | LINC00899 | TTS |
|  | 11 | 114086181 | 114086250 | 0.16 | 0.005 | 0.028 | ZBTB16 | Intron |
|  | 14 | 99241302 | 99241567 | -0.16 | 0.005 | 0.029 | BCL11B | Intron |
|  | 6 | 139540053 | 139540113 | 0.16 | 0.005 | 0.029 | LINC01625 | Intergenic |
|  | 20 | 58691884 | 58692203 | 0.16 | 0.005 | 0.031 | NPEPL1 | Promoter-TSS |
|  | 7 | 832456 | 832658 | -0.16 | 0.005 | 0.031 | SUN1 | Promoter-TSS |
|  | 7 | 77797160 | 77797221 | 0.16 | 0.005 | 0.031 | TMEM60 | Intron |
|  | 2 | 60358692 | 60358742 | 0.16 | 0.005 | 0.032 | MIR4432HG | TTS |
|  | 17 | 67441557 | 67441644 | -0.16 | 0.006 | 0.033 | MIR548AA2 | Intron |
|  | 16 | 85262602 | 85262917 | 0.16 | 0.005 | 0.033 | LINC00311 | Intergenic |
|  | 2 | 134305295 | 134305371 | -0.16 | 0.005 | 0.033 | MGAT5 | Intron |
|  | 12 | 113361856 | 113361888 | 0.16 | 0.006 | 0.034 | PLBD2 | Intron |
|  | 11 | 1849921 | 1850003 | 0.16 | 0.006 | 0.035 | LSP1 | Intergenic |
|  | 1 | 16156852 | 16157165 | 0.16 | 0.006 | 0.035 | EPHA2 | Promoter-TSS |
|  | 16 | 596253 | 596599 | 0.16 | 0.006 | 0.038 | RAB40C | Intron |
|  | 10 | 8059222 | 8059441 | -0.16 | 0.006 | 0.038 | GATA3 | Intron |
|  | 6 | 158660548 | 158660695 | -0.16 | 0.007 | 0.039 | SYTL3 | Intron |
|  | 11 | 48107516 | 48107553 | 0.16 | 0.007 | 0.039 | MIR3161 | Intron |
|  | 13 | 44412449 | 44412563 | 0.16 | 0.007 | 0.041 | TUSC8 | Intergenic |
|  | 7 | 100120297 | 100120405 | -0.16 | 0.007 | 0.042 | TAF6 | Promoter-TSS |
|  | 13 | 46898006 | 46898210 | -0.16 | 0.007 | 0.042 | HTR2A | Intergenic |
|  | 5 | 180684797 | 180684968 | -0.16 | 0.007 | 0.043 | LINC02222 | Non-coding |
|  | 16 | 30948272 | 30948354 | 0.16 | 0.007 | 0.044 | ORAI3 | Promoter-TSS |
|  | 11 | 67280606 | 67280839 | 0.16 | 0.007 | 0.044 | ANKRD13D | Intron |
|  | 7 | 128940217 | 128940297 | 0.16 | 0.008 | 0.046 | IRF5 | Promoter-TSS |
|  | 1 | 9893244 | 9893343 | 0.15 | 0.008 | 0.047 | CTNNBIP1 | Intron |
|  | 9 | 128106518 | 128106632 | -0.15 | 0.008 | 0.049 | SLC25A25 | Intron |
| **Urbanicity** |  |  |  |  |  |  |  |  |
|  | 12 | 131808785 | 131808956 | -0.18 | 0.001 | 0.005 | MMP17 | Intergenic |
|  | 5 | 141122080 | 141122232 | -0.17 | 0.002 | 0.009 | PCDHB4 | Exon |
|  | 2 | 88284077 | 88284414 | -0.17 | 0.002 | 0.010 | THNSL2 | Intergenic |
|  | 9 | 135105526 | 135105687 | 0.16 | 0.004 | 0.023 | OLFM1 | Intron |
|  | 5 | 141150057 | 141150897 | -0.15 | 0.004 | 0.025 | PCDHB6 | Exon |
|  | 2 | 16013824 | 16014598 | -0.15 | 0.005 | 0.032 | GACAT3 | Intergenic |
|  | 5 | 141199724 | 141200064 | -0.14 | 0.008 | 0.049 | PCDHB11 | Exon |
| **Total score** |  |  |  |  |  |  |  |  |
|  | 7 | 64885013 | 64885131 | -0.17 | 0.006 |  | ZNF273 | Intergenic |
|  | 8 | 78083944 | 78084046 | -0.17 | 0.006 |  | PKIA | Intergenic |
|  | 9 | 14910298 | 14910449 | -0.17 | 0.007 |  | FREM1 | Promoter-TSS |
|  | 1 | 29709864 | 29709904 | 0.17 | 0.007 |  | LINC01648 | Intergenic |
|  | 19 | 6372353 | 6372382 | -0.16 | 0.012 |  | ALKBH7 | Promoter-TSS |
|  | 1 | 179744491 | 179744695 | -0.15 | 0.016 |  | FAM163A | Intron |
|  | 16 | 3012597 | 3012654 | -0.15 | 0.020 |  | CLDN9 | Promoter-TSS |
|  | 13 | 100521364 | 100521527 | -0.14 | 0.022 |  | GGACT | Intron |
|  | 13 | 41193983 | 41194088 | -0.14 | 0.024 |  | KBTBD7 | Exon |
|  | 20 | 58839784 | 58839907 | -0.14 | 0.024 |  | GNAS | Promoter-TSS |
|  | 1 | 153609729 | 153610209 | 0.14 | 0.025 |  | S100A16 | Promoter-TSS |
|  | 11 | 66701618 | 66701746 | -0.14 | 0.026 |  | SPTBN2 | Exon |
|  | 7 | 156676403 | 156676487 | -0.14 | 0.029 |  | RNF32 | Exon |
|  | 1 | 153617759 | 153617891 | -0.14 | 0.029 |  | S100A14 | TTS |
|  | 8 | 101051561 | 101051716 | -0.14 | 0.031 |  | FLJ42969 | Promoter-TSS |
|  | 5 | 140862490 | 140863095 | -0.13 | 0.032 |  | PCDHA11 | 3' UTR |
|  | 10 | 132977804 | 132978088 | -0.13 | 0.035 |  | LINC01168 | Intergenic |
|  | 8 | 95073272 | 95073597 | -0.13 | 0.036 |  | MIR3150BHG | Promoter-TSS |
|  | 21 | 38957224 | 38957404 | 0.13 | 0.037 |  | LOC400867 | Intergenic |
|  | 2 | 16013824 | 16014598 | -0.13 | 0.038 |  | GACAT3 | Intergenic |
|  | 7 | 156942613 | 156942817 | -0.13 | 0.043 |  | NOM1 | Intergenic |
|  | 10 | 8059222 | 8059441 | -0.13 | 0.044 |  | GATA3 | Intron |
|  | 4 | 148261416 | 148261491 | 0.12 | 0.048 |  | NR3C2 | Intron |
|  | 22 | 37206964 | 37207014 | 0.12 | 0.048 |  | SSTR3 | Exon |

These results were adjusted for age, sex, DI, and CPZ equivalent (missing CPZ values were treated as 0).

Note: Chr: Chromosome; CPZ: Chlorpromazine; DI: Duration of illness; DMRs: Differentially Methylated Regions; FWER: Family-wise Error Rate; K-PERS: Korea Polyenvironmental Risk Score; r: Correlation Coefficient; SES: Socio-economic Status; TSS: Transcription Start Site; UTR: Untranslated Region.


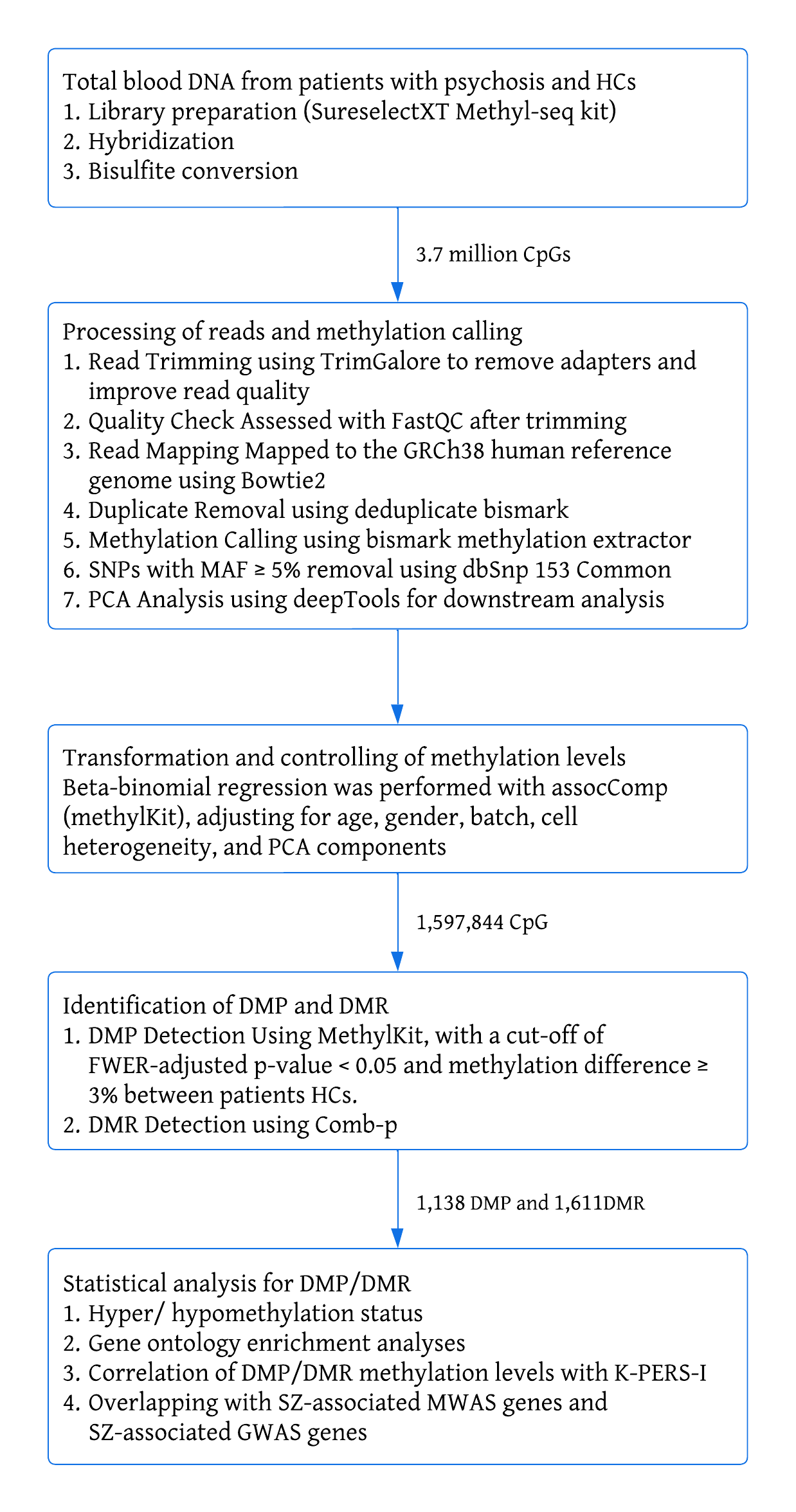


Fig S1. Analysis workflow.

Note: DMP, Differentially Methylated Position; DMR, Differentially Methylated Region; GWAS, Genome Wide Association Studies; HCs, Healthy Controls; MAF, Minor allele frequency; MWAS, Methylome-Wide Association Study; PCA, Principal Component Analysis; SNP, Single Nucleotide Polymorphism; SZ, Schizophrenia
